# A Network Based Model for Predicting Spatial Progression of Metastasis

**DOI:** 10.1007/s11538-025-01441-1

**Published:** 2025-04-09

**Authors:** Khimeer Singh, Byron A. Jacobs

**Affiliations:** 1 School of Computational and Applied Mathematics, University of the Witwatersrand, 1 Jan Smuts Avenue, Johannesburg, 2017 Gauteng South Africa; 2 Department of Mathematics and Applied Mathematics, University of Johannesburg, Auckland Park, PO Box 524, Johannesburg, 2006 Gauteng South Africa

**Keywords:** Mathematical oncology, Cancer biology, Computational modeling, Network modeling

## Abstract

Metastatic cancer is reported to have a mortality rate of 90%. Understanding the underlying principles of metastasis and quantifying them through mathematical modelling provides insights into potential treatment regimes. This work presents a partial differential equation based mathematical model embedded on a network, representing the organs and the blood vessels between them, with the aim of predicting likely secondary metastatic sites. Through this framework the relationship between metastasis and blood flow and between metastasis and the diffusive behaviour of cancer is explored. An analysis of the model predictions showed a good correlation with clinical data for some cancer types, particularly for cancers originating in the gut and liver. The model also predicts an inverse relationship between blood velocity and the concentration of cancer cells in secondary organs. Finally, for anisotropic diffusive behaviour, where the cancer experiences greater diffusivity in one direction, metastatic efficiency decreased. This is aligned with the clinical observation that gliomas of the brain, which typically show anisotropic diffusive behaviour, exhibit fewer metastases. The investigation yields some valuable results for clinical practitioners and researchers—as it clarifies some aspects of cancer that have hitherto been difficult to study, such as the impact of differing diffusive behaviours and blood flow rates on the global spread of cancer. The model provides a good framework for studying cancer progression using cancer-specific information when simulating metastasis.

## Introduction

Cancer is an illness that causes a loss of tissue homeostasis resulting in seemingly, uncontrollable cellular proliferation (Kuang et al. 2018). This disease is one of the leading causes of death worldwide. The global cancer burden stands at 19.3 million new cases, and almost 10 million deaths in 2020 (Ferlay 2021). Estimates show that around 90% of all cancer-related deaths occur due to metastasis (Weigelt et al. 2005)—a process characterised by the development of secondary tumours in regions away from the primary tumour. There are limited prediction and treatment capabilities for metastatic cancer because the process is a complex interplay between numerous physiological factors (Quaranta 2005). This paper attempts to elucidate aspects of metastasis by employing a network-based framework.

Mathematical modelling techniques can help us better understand the processes of cellular proliferation, metastasis and other related phenomena by abstracting the methods underlying the development of cancer. Insights obtained from these models may prove beneficial as many aspects of cancer, such as tumour growth and spread, can be challenging to monitor (Kuang et al. 2018).

There are various models in the literature for different components of cancer spread. These can range from one-dimensional differential equations which model tumour growth to hybrid simulations that model the local spread of cancer. For the one dimensional case, initial attempts were pioneered by Von Bertalanffy (1957), and Laird (1964). Later two-dimensional models incorporate Spatio-temporal effects, such as the model proposed by Gatenby and Gawlinski (1996). These principles were then expanded by Byrne (1996) to include different methods of cancer cell diffusion. The seminal model by Anderson (2000) factors in more of the histological facets of cancer.

Saidel et al. (1976) present an early attempt at modelling the local spread of cancer within the primary site. Regarding the use of hybrid modelling techniques, Quaranta et al. proposed an early example in Quaranta (2005). Of particular importance to this study is the work of Enderling (2006) and Franssen (2019), as these inform the work conducted in this investigation and, in the case of Franssen (2019), represent the most explicit attempt at modelling the spread of cancer to secondary sites using hybrid simulation techniques. Some atypical models are noted, for example, computational (Chen 2009) and Markov chain models (Liotta et al. 1976; Newton 2012, 2013) for their attempts to capture secondary spread.

The process of cancer growth and spread is a complex, multistep procedure. There are many models for the progression of tumours and the local spread of cancer cells. These provide helpful insight into the underlying dynamics of the steps in this process. However, models of secondary metastasis are still in their infancy—with very few explicitly catering for distant locations. Models that cater for this complexity can elucidate the process of metastasis for physicians and lead to better prediction capabilities for metastatic cancer. A better understanding of metastasis may lead to more optimised treatment, whilst improved predictive capabilities can provide more realistic outlooks to patients. It is therefore crucial that we further investigate metastatic models.

Given the scarcity of such models, this study provides a framework capable of modelling metastasis in a spatially exact manner that can help clarify aspects of metastasis. Evaluating the model against clinical data shows that the model produces realistic results. Currently, very few models account for the spread to secondary locations. We address this shortage by providing a method that generically models metastasis. The technique can determine likely secondary sites based on any primary site covered in the study. We also use the framework to simulate patient and cancer specific phenomena, namely the velocity of blood and diffusive behaviour of the cancer. Allowing us to study the impact of cancers which may deviate from clinically observed patterns.

## Dynamics of Metastasis

The focus of this study is the metastatic spread of cancer between organs on a vascular network. A review of the underlying biological processes that govern this phenomenon follows. Of particular interest is the invasion-metastasis cascade. This sequence refers to the ordered chain of events required for metastasis to occur (Wittekind and Neid 2005).

### Local Cell Dynamics

Human tissue is composed of cells, most of which contain the genomic information of the entire organism; this allows for continuous cellular repair and reproduction. Occasionally, a cell may access unnecessary information for the tasks mentioned above. This aberration may alter the proliferation rate of the cell and hence form a tumour through uncontrolled cellular proliferation (Weinberg 2006). While this mechanism facilitates tumorigenesis, further steps are needed for the tumour to evolve into cancer. Hanahan and Weinberg outline these steps in Hanahan and Weinberg (2000). We present a summary of these steps:

1. Self-sufficient growth: the tumour can proliferate.
2. Insensitivity to growth suppression: the existing controls to mitigate proliferation are rendered inert.
3. Evasion of apoptosis: cells do not die off as quickly.
4. Limitless replicative potential: no factors limit cell growth.
5. Procurement of blood vessels via angiogenesis: once a tumour reaches a significant size, it requires a blood supply to provide it with oxygen and nutrients. The formation of this vascular network is known as angiogenesis (Nishida 2006).
6. Acquisition of metastatic properties: the tumour assumes the necessary characteristics for metastasis to occur.

We summarise the process of angiogenesis and the metastatic cascade in the proceeding section.

### Invasion Metastasis Cascade

An estimated $40\%$ of metastasis spreads via the blood vessels; so-called hematogenous metastasis (Font-Clos et al. 2020). Another important driver of metastasis is lymphatic spread, which is the transfer of cells via lymph. Lymphatic spread is currently beyond the scope of this study, as we focus on hematogenous spread. The invasion-metastasis cascade refers to the sequence of events that precede hematogenous metastasis. For a tumour to metastasise, it must first produce blood vessels via angiogenesis. We begin with a brief explanation of how tumours interact with their surrounding medium-thus enabling us to explore metastasis in greater depth.

Tumours begin when epithelial cells start to proliferate. As they grow, the tumour invades the layer directly underneath the epithelial cells, known as the basal lamina. The cancer cells then migrate through the extra-cellular matrix (ECM), the connective tissue between cells. The cancer cells release special enzymes to degrade the matrix and facilitate migration, these are generally called matrix degrading enzymes (MDEs). The tumour releases growth factors that aid in developing new blood vessels and capillaries. The newly formed blood vessels connect to existing vascular structures to supply the tumour with blood; this also provides the tumour with an opportunity to enter the bloodstream, as the new vasculature is often poorly formed. Material from the tumour can thus enter by flowing into these structures (Cooper et al. 2007).

The first step in the invasion metastasis cascade is local invasion. Which is the process of cancer cells detaching from the primary tumour and invading the surrounding ECM. From here, the tumour cells can enter the bloodstream through the second step, known as intravasation. Intravasation occurs when tumour cells migrate into existing vascular structures. Cancer cells can enter these vessels by passing through the vessel wall or poorly constructed vessels formed through angiogenesis. Thereafter the cells must survive within the circulatory system. Upon entering the bloodstream, the cells face several stresses. Some of these stresses include apoptosis triggered by the loss of connection to a substrate, damage to the cell due to hemodynamic shear forces and predation by immune cells. Should the cells survive in the blood stream, they may extravasate. Extravasation is when cancer cells exit the vascular system. Theoretically, tumour cells should colonise any other organ in the body. However, the cells usually metastasise on a small subset of organs. A potential explanation for this is the tumours’ inability to enter the micro-vessels of specific organs. If the diameter of the micro-vessels at these sites is small enough, the cells cannot enter the circulatory system of that organ. If a cell does enter these micro-vessels, it may extravasate by rupturing the vessel walls. The final step in the cascade is colonisation. If the cells survive in their new environment post-extravasation, they are still not guaranteed to proliferate. Tumour cells must adapt to their newfound surroundings and the host of challenges therein, such as inflammatory responses that release anti-angiogenic factors. If a tumour does manage to proliferate in the new environment and reaches a clinically detectable size, it is considered a “metastases". The tumour has successfully colonised the organ and concluded the invasion-metastasis cascade.

### The Seed and Soil Hypothesis

English physician Stephen Paget proposed the seed and soil hypothesis (Paget 1889). Paget presented the hypothesis as a potential explanation for why disseminated cancer cells prefer specific organs. After conducting autopsies on women with fatal breast cancer, he noted a discrepancy between the organs that cancer spread to and the relative blood supply of these regions (Fidler and Poste 2008). This phenomenon led him to conclude that cancer cells arising in specific organs (seeds) require a hospitable environment to proliferate (soil) (Paget 1889). Hence, cancers may favour particular metastatic sites, depending on the primary site. This proposal still holds today (Fidler 2003)—however; it cannot explain the metastatic patterns of all cancers (Weinberg 2006).

## Arrangement of Model

This section provides an overview of the models that govern cancer growth, the local spatial distribution, and the transport of cells through the vasculature. In the case of cancer growth and dispersion, we determine the general behaviour of cancer cells on a single organ. Regarding vascular transport, we explore the mechanics that underlie the cancer cells’ motion as they flow within the bloodstream.

The models used in this investigation are constructed from the frameworks depicted in Franssen (2019) and Pera et al. (2019). The agent-based model from Franssen (2019), which was primarily responsible for the reproduction of cancer cells, is relaxed to save on computational cost. In its place, the system illustrated in Pera et al. (2019) compensates for the growth of cancer. Using this system and its associated diffusion tensor, we can study the impact of different diffusive behaviours on metastasis.

Advection-decay equations control the transport of cancer cells through the vascular network. The cells move in accordance with the velocity of blood in that particular vessel. Since the cell is robbed of the nutrients it would typically obtain from the surrounding organ and is exposed to shear stress in the bloodstream, it is prone to decay—hence the inclusion of a decay term. Here we derive the equation and adapt it to a network architecture.

### Modelling Local Spread

We consider the model defined by Enderling (2006) and later expanded upon by Pera et al. (2019). The model, is reproduced to simulate organ-level dynamics of a growing tumour. The non-dimensional form of the equation is expressed as:

1 $$\begin{aligned} \frac{\partial n}{\partial t}&= \overbrace{\mu n (1 - n - f)}^{\textit{proliferation}} + \overbrace{\mathbb {D} \nabla ^2 n}^{\textit{diffusion}} - \overbrace{\chi \nabla \cdot (n \nabla f)}^{\textit{haptotaxis}}, \nonumber \\ \frac{\partial m}{\partial t}&= \overbrace{D_m \nabla ^2 m}^{\textit{diffusion}} + \overbrace{\zeta n (1 - m)}^{\textit{production}} - \overbrace{\omega m}^{\textit{decay}}, \nonumber \\ \frac{\partial f}{\partial t}&= \overbrace{-\kappa m f}^{\textit{decay}}. \end{aligned}$$

where *n*, *m* and *f* refer to the cancer cell, MDE and ECM concentrations respectively. The system is defined on the unit square $\Omega = (0, 1) \times (0, 1)$. Cancer cell death is not catered for by the model. The risk of not accounting for cell death is the potential for unconstrained growth. This risk is mitigated in the model, as the proliferation rate is limited by the available “free space" in the domain, i.e, the $(1 - n - f)$ term. Furthermore, in environments which are more stressful for the cell than organs (such as the blood stream) cell death is explicitly catered for. The growth model is selected for simplicity, despite not capturing some of the complexities of tumour growth, as the focus of the model is on how a growing tumor can metastasize.

Notably, we have kept the diffusion tensor:

2 $$\begin{aligned} \mathbb {D} = D_n \begin{pmatrix} a(x, y) & b(x, y) \\ b(x, y) & c(x, y) \end{pmatrix}. \end{aligned}$$

where *a*, *b* and *c* are functions of space and $D_n$ is the diffusive constant of *n*. Consideration for non-standard diffusion accounts for the atypical diffusive behaviour which some tumours exhibit. As per the methodology in Enderling (2006) and Pera et al. (2019), we apply zero-flux boundary conditions to our solution. The boundary conditions are:

3 $$\begin{aligned} \begin{aligned} n \cdot \hat{v}&= 0, \\ f \cdot \hat{v}&= 0, \\ m \cdot \hat{v}&= 0. \end{aligned} \end{aligned}$$

where $\hat{v}$ is the outer unit normal. These conditions hold on the boundary of $\Omega$, which is defined as $\partial \Omega$. The initial conditions for the system are given by:

4 $$\begin{aligned} \begin{aligned} n(x, y, 0)&= N_0 e^{-\omega _0((x - 0.5)^2 + (y - 0.5)^2)}, \\ m(x, y, 0)&= \frac{1}{2} n_0, \\ f(x, y, 0)&= 1 - \frac{1}{2} n_0. \end{aligned} \end{aligned}$$

With $N_0 = 0.75$ and $\omega _0 = 0.005$, as per the values outlined by Pera et al. (2019). These conditions allow us to create a radial tumour in the centre of the domain.

### Advection Decay Equation

Blood carries the cancer cells after they enter the bloodstream. The conditions in the bloodstream differ significantly from the primary tumour site, resulting in a more hostile environment for the cells. Some factors which reduce the survival of cells within the vessels include hydrodynamic shear forces, which can tear the cell apart, and the lack of a substrate for the cell to latch onto Weinberg (2006).

The framework defined in by Heaton (2012) is applied to accommodate the situation; this entails defining an advection equation that governs the flow of particles in fluid over a network architecture. An advection equation adequately represents the transport component of this process. A decay term is appended to the advection equation to account for the hostility of the vascular environment. The equation assumes the form:

5 $$\begin{aligned} \frac{\partial n_{ij}}{\partial t} = \overbrace{v \frac{\partial n_{ij}}{\partial x}}^{\textit{Advection}} - \overbrace{\alpha n_{ij}}^{\textit{Decay}}. \end{aligned}$$

where $n_{ij}$ refers to the number of cancer cells in a blood vessel between organ nodes *i* and *j*, *v* denotes the velocity of blood and $\alpha$ signifies the death rate of cells in the vasculature. The domain for the equation is the one-dimensional vessel *i*, *j* between nodes *i* and *j*.

### Adaption to Network Architecture

#### Organ-Vasculature Coupling

A simplified representation of the circulatory system is introduced to replace the multi-grid approach employed by Franssen (2019). This representation is based on the quantity of blood that certain major organ systems receive. In particular, these are the brain, lung, liver, gut and kidney. These organs were selected because they account for a significant proportion of secondary metastases sites.

**Fig. 1:**
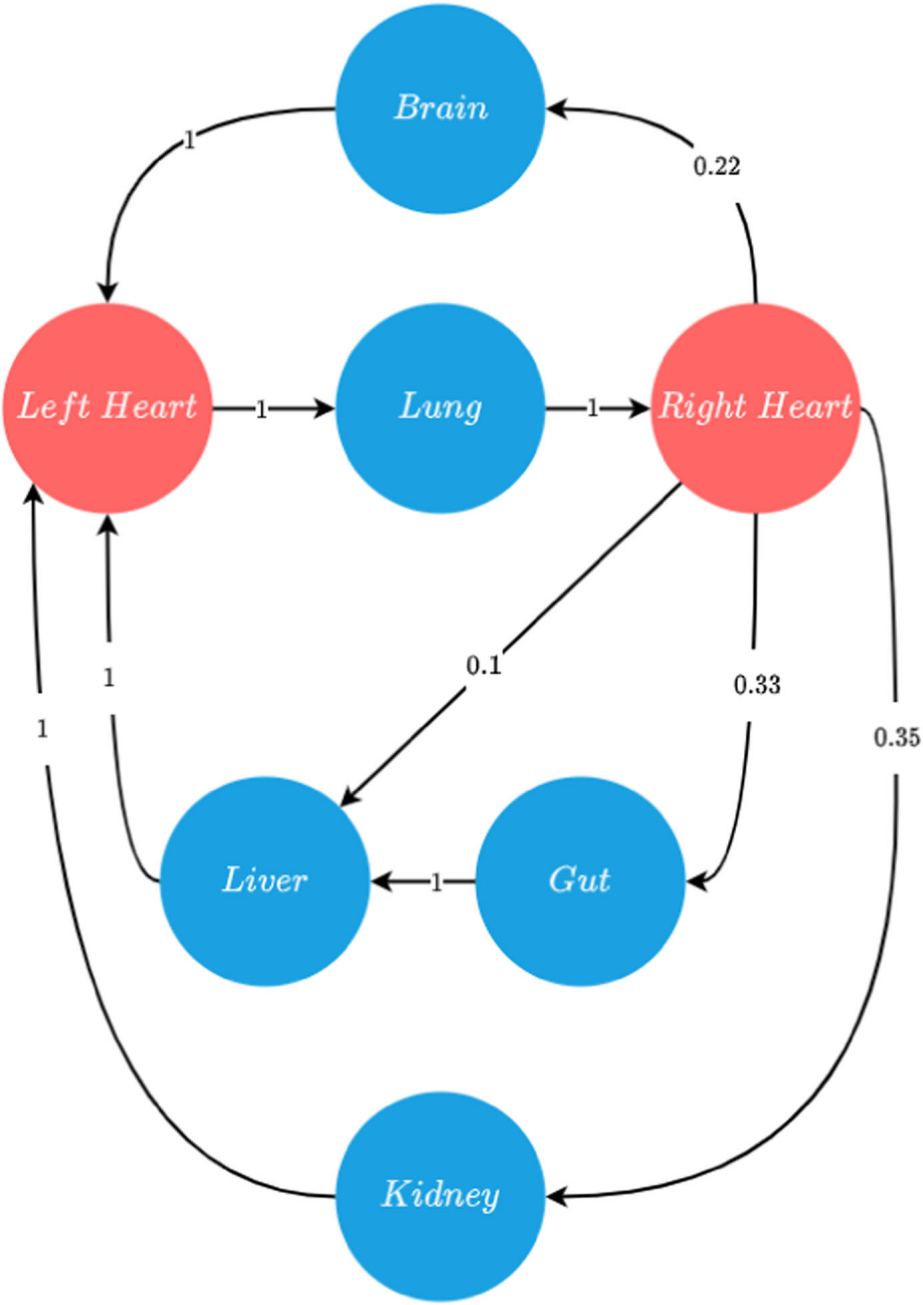
Simplified model of human circulatory system displaying cardiac output to various organ systems at a given point in time, adapted from Iaizzo (2010). Blue nodes indicate organ systems, while red nodes are chambers of the heart. The edge weightings denote the proportion of blood leaving the organ (Color figure online)

The circulatory pathways between these organs are well understood from a biological perspective, for example, oxygenated blood flows from the right chamber of the heart to the brain via the carotid artery. In order to capture this structure, the system is arranged as a weighted directed graph. Each node corresponds to an organ and each edge corresponds to a blood vessel that carries blood between organs. These edges are the main arteries and veins that transfer blood between these systems. The edges of the graph are weighted by the proportion of blood that moves from the out-going node to the incoming node. This proportionality allows us to bias the quantity of cancer cells that various organ systems receive according to how much blood they are effused with. These weights, and the structure of the graph itself, are inferred from the simplified model of circulation described by Iaizzo (2010). The full graph is displayed in Fig. 1. Notably, the heart is decomposed into the left and right chambers, as each chamber acts as a separate node, with the left chamber receiving de-oxygenated blood from the various organ systems via veins and the right chamber sending oxygenated blood to the organs via arteries.

The length of the vascular pathway between organ systems is needed to model the advection of cancer cells within these vessels. Since each edge corresponds to a major artery or vein, the length of this is taken as the distance between the organ nodes.

As this approach represents a simplified model, there is some nuance that is sacrificed at the expense of simulability. For example, it is known that blood vessels branch out and distribute blood through a network of capillaries. The main impact this has on the movement of cancer cells is the entrapment of cells within these sub-vessels as the arteries begin to narrow. These dynamics can impact how many cells enter the organ. To account for this, a monte-carlo simulation is established to determine what proportion of cells are trapped and which enter the organ. This is elucidated in Sect. 3.3.4.

Figure 1 is based on the system provided by Iaizzo (2010), which provides a view of the volume of blood the various organ systems in the body receives. The quantity of cancer cells delivered to an organ is biased in proportion to the organ’s blood supply. Figure 1 serves as a digraph that we use to represent the vascular system. The arteries, which carry oxygenated blood from the heart, and the veins, which carry deoxygenated blood to the heart, are expressed as directed edges of varying lengths. Each vertex is weighted per the proportion of blood the organ system receives. We have excluded other locations, such as bone and skin, to focus on the major organ systems. Furthermore, these organs have more data to inform our analysis.

The digraph formed from Fig. 1 admits the vertex set *V*:

6 $$\begin{aligned} V = \{\textit{lung}, r. \, \textit{heart}, l. \, \textit{heart, brain, gut, liver, kidneys}\}, \end{aligned}$$

where $r. \, heart$ and $l. \, heart$ refer to the chambers of the heart that distribute oxygenated blood and receive de-oxygenated blood respectively. The corresponding edge set *E* contains all arteries and veins under study:

7 $$\begin{aligned} \begin{aligned} E = \{\{r. \, \textit{heart, lung}\}, \{\textit{lung}, l. \, \textit{heart}\}, \{l. \, \textit{heart, brain}\}, \\ \{l. \, \textit{heart, gut}\}, \{l. \, \textit{heart, kidney}\}, \{\textit{gut, liver}\}, \\ \{\textit{kidney}, r. \, \textit{heart}\}, \{\textit{liver}, r. \, \textit{heart}\}, \{\textit{brain}, r. \, \textit{heart}\}\}. \end{aligned} \end{aligned}$$

The following Table 1 describes the arteries and veins corresponding to the network’s edges. We also provide the estimated length of the vessel. The lengths are not indicative of the actual distance between these sites, as many smaller vessels were excluded from the study. However we consider the main artery or vein supplying the organ to be a reasonable approximation for the actual distance. We use the length of its corresponding vessel where lengths are unavailable for a particular vein or artery. For example, we use the length of the carotid artery as an estimate for the jugular, as one is the artery that supplies the organ, and the other is the vein that takes blood away from it. We also include the cardiac outputs from Fig. 1 as these serve as our weights for the network. The vessel length and blood velocity technically serve as weights; however, we do not display them to maintain a clear and concise notation.

**Table 1:** Blood vessel output and lengths

Edge	Vessel type	Vessel name	Cardiac output	Vessel length (cm)	Reference
{*r. heart, lung*}	Artery	Pulmonary Artery	1	5	Tucker et al. (2020)
{*lung, l. heart*}	Vein	Pulmonary Vein	1	5	Tucker et al. (2020)
{*l. heart, brain*}	Artery	Carotid	0.22	20	Choudhry (2016)
{*l. heart, gut*}	Artery	Abdominal Aorta	0.33	13	Gameraddin (2019)
{*l. heart, kidney*}	Artery	Renal Artery	0.35	3	Müller and Toro (2014)
{*l. heart, liver*}	Artery	Hepatic Artery	0.10	6.5	Müller and Toro (2014)
{*gut, liver*}	Vein	Hepato Portal Vein	1	6.80	Müller and Toro (2014)
{*kidney, r. heart*}	Vein	Renal Vein	1	8.5	Cope and Isard (1969)
{*liver, r. heart*}	Vein	Hepatic Vein	1	6.80	Müller and Toro (2014)
{*brain, r. heart*}	Vein	Jugular	1	20	Choudhry (2016)

Since all the blood in each organ goes back to the heart, the cardiac output of every vein is 1.

We denote the weight of an edge between node *i* and *j* as $\gamma _{i, j}$. This weighting corresponds to the proportion of blood that the organ represented by *i* sends to the organ represented by *j*. The graph thus assumes the following adjacency matrix:

8 $$\begin{aligned} A = \left[ \begin{array}{c|ccccccc} & \textit{lung} & l. \, \textit{heart} & r. \, \textit{heart} & \textit{brain} & \textit{gut} & \textit{liver} & \textit{kidney} \\ \hline \textit{lung} & 0 & 1 & 0 & 0 & 0 & 0 & 0 \\ l. \, \textit{heart} & 0 & 0 & 0 & 0.22 & 0.33 & 0.1 & 0.35 \\ r. \, \textit{heart} & 1 & 0 & 0 & 0 & 0 & 0 & 0 \\ \textit{brain} & 0 & 0 & 1 & 0 & 0 & 0 & 0 \\ \textit{gut} & 0 & 0 & 0 & 1 & 0 & 1 & 0 \\ \textit{liver} & 0 & 0 & 1 & 0 & 0 & 0 & 0 \\ \textit{kidney} & 0 & 0 & 1 & 0 & 0 & 0 & 0 \\ \end{array}\right] \end{aligned}$$

Adapting the system of equations defined in Pera et al. (2019) to this domain is done by implementing the equations across all nodes. The system then takes the form:

9 $$\begin{aligned} \begin{aligned} \frac{\partial n_i}{\partial t}&= \mu n_i \left( 1 - \frac{n_i}{n_0} - \frac{f_i}{f_0}\right) + \mathbb {D}_i \nabla ^2 n_i - \chi \nabla \cdot (n_i \nabla f_i) - \sum _{j, j \ne i}^{v_i} \frac{E_i}{\gamma _{ij}},\\ \frac{\partial m_i}{\partial t}&= D_m \nabla ^2 m_i + \zeta n_i \left( 1 - \frac{m_i}{m_0}\right) - \omega m_i, \\ \frac{\partial f_i}{\partial t}&= -\kappa m_i f_i.\\ \end{aligned} \end{aligned}$$

where $i \in V$ and $n_i$, $m_i$ and $f_i$ refer to the cancer cell, MDE and ECM concentration at node *i*. The summation term accounts for the movement of cells outside of node *i*. Here $v_i$ is a subset of *V* and contains all the nodes that cells can travel to from node *i*. The term $E_i$ refers to the portion of cells which have intravasted from *i* and is defined in the proceeding section. For simplicity, the constants remain the same across all nodes. The system persists zero-flux boundary conditions as per (3) With initial conditions aligned with (4). The primary node refers to the node at which cancer originates and is the node on which the initial conditions are applied. Each node is modelled as a $128 \times 128$ grid to allow for numerical simulation of the equations and facilitate other simulation dynamics, such as extravasation through blood vessels. This grid size was selected to maintain consistency with Pera et al. (2019). For cancer to spread to the other nodes, it must travel through a vascular section. A review of this process follows.

#### Intravasation

On each grid, eleven random points are chosen as blood vessels through which the cancer cells can exit and enter. This number of vessels were selected to be consistent with Franssen (2019), where ten points were selected. The number of vessels has been scaled up since the grid is slightly larger. For each simulation iteration, the cancer cells located on a blood vessel enter the bloodstream. The total number of cells exiting a node *i* is:

10 $$\begin{aligned} E_i = \epsilon \sum _{b=1}^{11} n_i(x_b, y_b). \end{aligned}$$

where $n_i(x_b, y_b)$ represents the number of cells contained on a blood vessel point $(x_{b}, y_{b})$ on the node *i* and $\epsilon$ is the proportion of cells at the vessel which successfully intravasate. This quantity is accumulated from all the blood vessel points on the node. We set $\epsilon$ to $4.5 \times 10^{-6}$, in line with proportions observed by Kim (2016).

#### Vascular Transport

Blood carries the cancer cells after they enter the bloodstream. The conditions in the bloodstream differ significantly from the primary tumour site, resulting in a more hostile environment for the cells. Some factors which reduce the survival of cells within the vessels include hydrodynamic shear forces and the lack of a substrate which the cell can latch onto Weinberg (2006).

We use the Eq. (5) that we defined earlier to model advection through the vasculature. Since $\alpha$ signifies the death rate of cells in the vasculature, we let $\alpha = 5.8 \times 10^{-6}$. This value is obtained from work conducted by Liu et al. (2014). Here, they study the effect of hemodynamic shear stress on cancer cells in circulation. They surmise that roughly $50\%$ of cancer cells die every 24 h. We arrive at our chosen value by expressing this rate in seconds. The following boundary condition is prescribed:

11 $$\begin{aligned} n_{i,j}(0, t) = \frac{E_{i}}{\gamma _{i, j}}. \end{aligned}$$

where $E_i$ is the above mentioned exit proportion at node *i* and $\gamma _{i, j}$ is the weighting of the edge between *i* and *j*. This equation implies that the number of cells leaving node *i* serves as the boundary condition for the transport system. Therefore, at each simulation iteration, several cells proportional to the concentration at node *i* will enter the bloodstream. These cells divide across all the outgoing blood vessels connected to the organ. The amount of cells each vessel receives is proportional to the volume of blood that the corresponding organ receives. This process is visualised in the following figure.

**Fig. 2:**
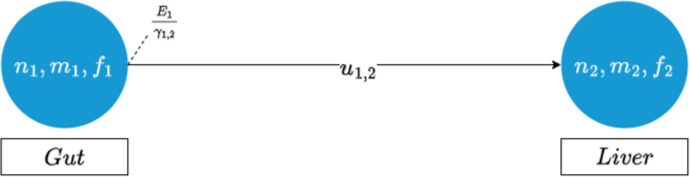
An overview of the transport dynamics between the gut and liver. Here, $u_{ij}$ refers to the edge between node *i* and *j*. The cells will be subject to the system defined in 5

In Fig. 2, we highlight one of the main contributions of this work. Here, we describe the transport dynamics along the edges through the auxiliary equation (5). The equation is coupled to the system of Eq. (9) at the vertices through the boundary conditions.

#### Extravasation

Cancer cells colonise other organs through the blood vessels. The process of extravasation can occur at all organ systems, except the left and right heart nodes as these represent chambers of the heart and are used to simulate circulatory mechanics accurately. The cells contained at the point $n_{ij}(L, t)$ are distributed into the node *j* on a randomly chosen blood vessel point. To accurately simulate metastatic invasion, a proportion of cells are trapped in the micro-circulation of the organ. Some of these cells are lethally damaged in this location and thus do not extravasate. The remainder stays in the circulatory system and enter the heart via the corresponding vein. A value for the proportion of cells that stay in the circulatory system could not be obtained from the literature. In this section we provide an estimation for this value.

Monte Carlo methods are well suited to solving problems involving variables that are seemingly random. Typically, a Monte Carlo simulation involves sampling a statistical distribution to generate random values for these variables. This simulated random data can be used to draw conclusions about the underlying processes.

A Monte Carlo simulation was conducted to estimate the proportion of cells that remain trapped in the circulatory system, which we designate as $\kappa$. The simulation compares the diameter of the capillaries that cells pass through against the diameter of circulating tumour cells. After running this comparison for a certain number of iterations, we estimate a value for $\kappa$. Upper and lower bounds for the diameters were sourced from the relevant literature. These ranges and the associated references are visualised in the Table 2 below.

**Table 2:** Tumour cell and capillary diameter ranges

Variable	Lower bound (*LB*)	Upper bound (*UB*)	Reference
Tumour cell diameter ($T_d$)	7.2	15	Harouaka et al. (2013)
Capillary diameter ($C_d$)	5	10	Iaizzo (2010)

To correctly simulate this scenario, we generate random observations for the diameter of capillaries and tumour cells using the prescribed ranges from the preceding table. Since we lack substantial empirical data for these properties, we must use estimates to obtain some of the values needed to create these observations—in particular, the mean and standard deviation of data that would fall within this range. We use the following method prescribed by Wan (2014) to arrive at this estimate:

12 $$\begin{aligned} \bar{X}&= \frac{UB + LB}{2}, \end{aligned}$$

13 $$\begin{aligned} \sigma&= \frac{UB - LB}{\sqrt{12}}. \end{aligned}$$

where $\bar{X}$ is the mean and $\sigma$ is the standard deviation. It is assumed that these values are log-normally distributed since they cannot assume a negative value. Using a log-normal random number is also more realistic than a uniform random observation, as extreme values are less likely in the log-normal case. We thus create an estimated value for the diameter of CTCs and capillaries by inverse sampling a log-normal distribution with a mean and standard deviation provided by Eqs. (12)–(13). For each simulation iteration, a random value is generated, giving us a simulated cancer cell and capillary diameter. If the cancer cell is too large for the capillary, the cell remains in the micro-circulation of the organ. If the cell is smaller than the capillary, it passes through the organ and remains in circulation. While this method only provides a rough approximation, it does account for the large degree of variability displayed in blood vessels and tumour cell sizes whilst remaining within a reasonable range.

**Fig. 3:**
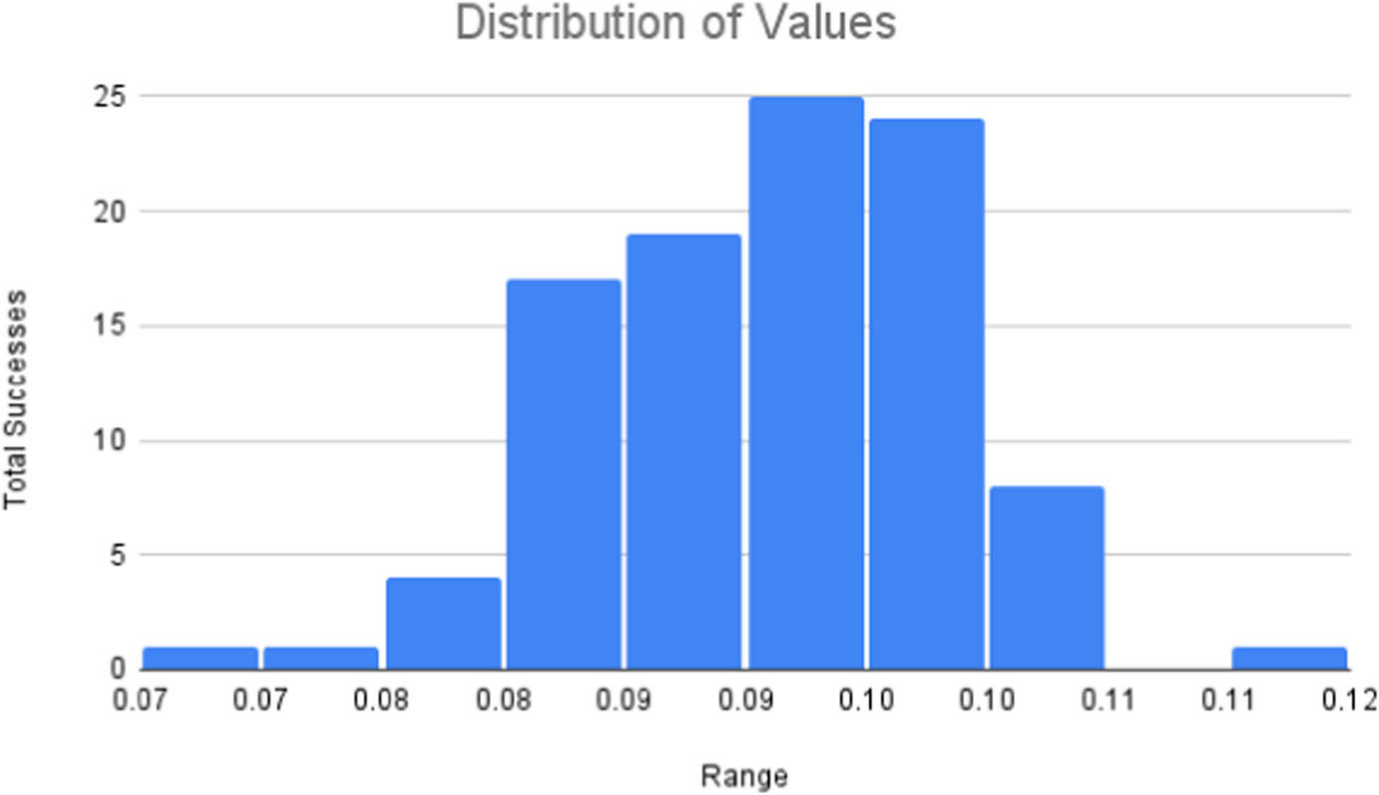
Distribution of $\kappa$-values

**Fig. 4:**
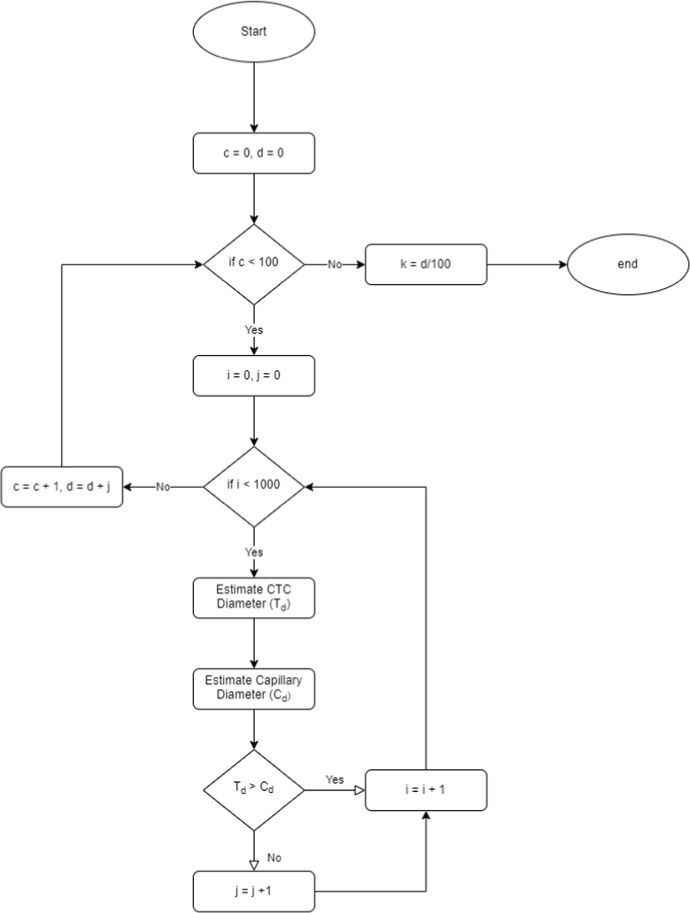
Monte Carlo simulation algorithm

**Fig. 5:**
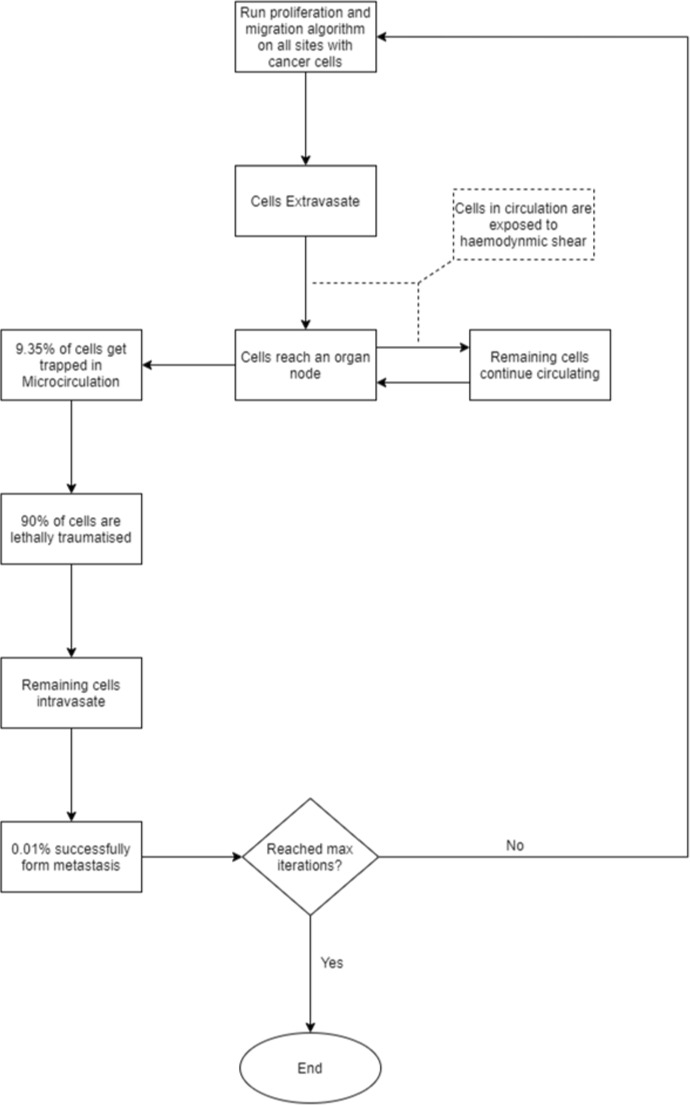
Overview of full network based simulation

The simulation ran for 10000 iterations. This value is selected as it was experimentally observed that the simulation converged to our final $\kappa$ value after approximately 10000 iterations. By counting the iterations which resulted in a cell passing through the capillaries and dividing by 10000, we obtain values for $\kappa$. We repeat this process 100 times to produce a distribution of $\kappa$-values. In line with the observed results, $\kappa$ is selected as 0.0935. This value corresponds to the average of the most common range in the distribution from Fig. 3. The simulation mechanics for the Monte Carlo simulation is summarised in Fig. 4. An overview of the overall network based simulation mechanics is provided in Fig. 5

### Numerical Simulation of Model

#### Numerical Scheme Applied to Cancer Model

Considering the system (1), we apply a second order central difference discretisation to approximate the spatial derivative and an explicit Euler method to calculate the time derivative. The second order central difference scheme for the spatial derivatives are stated as:

14 $$\begin{aligned} u_{xx}&= \frac{u_{i+1, j}^{k} - 2u_{i, j}^{k} + u_{i-1, j}^{k}}{\Delta x^2}, \nonumber \\ u_{yy}&= \frac{u_{i, j+1}^{k} - 2u_{i, j}^{k} + u_{i, j - 1}^{k}}{\Delta y^2}, \nonumber \\ u_{xy}&= \frac{u_{i+1, j+1}^{k} - u_{i + 1, j - 1}^{k} + u_{i - 1, j + 1}^{k} + u_{i-1, j+1}^{k}}{\Delta xy}. \end{aligned}$$

The explicit Euler method for the time derivative is given as:

15 $$\begin{aligned} u_{t} = \frac{u_{i, j}^{k + 1} - u_{i, j}^{k}}{\Delta t}. \end{aligned}$$

The domain is discretised as an $\Omega = (0, 1) \times (0, 1)$ unit square on the Cartesian plane. A coordinate (*x*, *y*) is thus represented by $(i \Delta x, j \Delta y)$, where $i = 0, 1,\ldots , L_{1}$ and $j = 0, 1,\ldots , L_{2}$ with $L_{1}, L_{2} \in \mathbb {N}$. Furthermore, $\Delta x, \Delta y$ denote the space steps in the *x* and *y* direction respectively. The discrete form of the time variable is $k \Delta t$, where $\Delta t$ is the constant time step and $k \in \mathbb {N}$. The variables *n*, *f* and *m* are thus approximated at the grid points (*i*, *j*) for discrete time step $k \Delta t$ as follows,

16 $$\begin{aligned} \begin{aligned} N^{k}_{i, j}&\approx n(i \Delta x, j \Delta y, k \Delta t), \\ F^{k}_{i, j}&\approx f(i \Delta x, j \Delta y, k \Delta t), \\ M^{k}_{i, j}&\approx m(i \Delta x, j \Delta y, k \Delta t). \end{aligned} \end{aligned}$$

Furthermore, the diffusion tensor assumes the following discrete form,

17 $$\begin{aligned} \begin{aligned} a_{i, j}&= a(i \Delta x, j \Delta y), \\ b_{i, j}&= b(i \Delta x, j \Delta y), \\ c_{i, j}&= c(i \Delta x, j \Delta y). \end{aligned} \end{aligned}$$

We discretise the system (1) using the derivatives outlined in Eqs. (14)–(15) and the terms specified in (16) and (17) to obtain the following numerical scheme:

18 $$\begin{aligned}&\begin{aligned} N^{k+1}_{i, j}&= N^{k}_{i, j} + a_{i, j} \frac{\Delta t}{\Delta x^2}(N^{k}_{i + 1, j} - 2N^{k}_{i, j} + N^{k}_{i - 1, j}) \\&\quad + c_{i, j} \frac{\Delta t}{\Delta y^2}(N^{k}_{i, j + 1} - 2N^{k}_{i, j} + N^{k}_{i, j - 1}) \\&\quad + b_{i, j} \frac{\Delta t}{2 \Delta x \Delta y} (N^{k}_{i + 1, j + 1} - N^{k}_{i + 1, j - 1} - N^{k}_{i - 1, j - 1} + N^{k}_{i - 1, j - 1}) \\&\quad + \frac{\Delta t}{4 \Delta x^2} (a_{i + 1, j} - a_{i - 1, j})(N^{k}_{i + 1, j} - N^{k}_{i - 1, j}) \\&\quad + \frac{\Delta t}{4 \Delta y^2} (c_{i, j+1} - a_{i, j - 1})(N^{k}_{i, j + 1} - N^{k}_{i, j - 1}) \\&\quad + \frac{\Delta t}{4 \Delta x \Delta y} [(b_{i + 1, j} - b_{i - 1, j})(N^{k}_{i, j + 1} - N^{k}_{i, j - 1}) + (b_{i, j + 1} - b_{i, j - 1})(N^{k}_{i + 1, j} - N^{k}_{i - 1, j})] \\&\quad + \gamma \frac{\Delta t}{4 \Delta x^2}(N^{k}_{i + 1, j} - N^{k}_{i - 1, j})(F^{k}_{i + 1, j} - F^{k}_{i - 1, j}) \\&\quad + \gamma \frac{\Delta t}{4 \Delta y^2}(N^{k}_{i, j + 1} - N^{k}_{i, j - 1})(F^{k}_{i, j + 1} - F^{k}_{i, j - 1}) \\&\quad + \gamma \frac{\Delta t}{4 \Delta x^2} N^{k}_{i, j}(F^{k}_{i + 1, j} - 2F^{k}_{i} + F^{k}_{i - 1, j}) \\&\quad + \gamma \frac{\Delta t}{4 \Delta y^2} N^{k}_{i, j}(F^{k}_{i, j + 1} - 2F^{k}_{i} + F^{k}_{i, j - 1}) \\&\quad + \Delta t \lambda N^{k}_{i, j} (1 - N^{k}_{i, j} - F^{k}_{i, j}), \end{aligned}\nonumber \\&\begin{aligned} F^{k+1}_{i, j}&= F^{k}_{i, j} (1 - \Delta t \kappa M^{k}_{i, j}), \end{aligned}\nonumber \\&\begin{aligned} M^{k+1}_{i, j}&= M^{k}_{i, j} + d_{m} \frac{\Delta t}{\Delta x^{2}}(M^{k}_{i + 1, j} - 2M^{k}_{i, j} + M^{k}_{i - 1, j}) \\&\quad + d_{m} \frac{\Delta t}{\Delta y^{2}}(M^{k}_{i, j + 1} - 2M^{k}_{i, j} + M^{k}_{i, j - 1})\\&\quad + \Delta t \delta N^{k}_{i, j} (1 - M^{k}_{i, j}) - \Delta t \beta M^{k}_{i, j}. \end{aligned} \end{aligned}$$

The numerical scheme is selected because of its ease of implementation and because the stability of the scheme is guaranteed, provided the following upper bound on the time step, $\Delta t$, is preserved (Pera et al. 2019):

19 $$\begin{aligned} \Delta t \le min \left\{ \frac{1}{8} \frac{max(\Delta x^2, \Delta y^2)}{max_{i, j}(a_{i, j}, c_{i, j})}, \frac{1}{2} \frac{max(\Delta x^2, \Delta y^2)}{d_m} \right\} . \end{aligned}$$

#### Numerical Scheme Applied to Advection Equation

In order to solve equation 3.1, a first order upwind scheme is applied. This method considers the direction in which a substance is propagated through the domain. For our purposes, the substance is blood flowing through vessels. The scheme assumes the form:

20 $$\begin{aligned} &  \begin{aligned} \frac{u^{k + 1}_{i} - u^{k}_{i}}{\Delta t} + a\frac{u^{k}_{i} - u^{k}_{i-1}}{\Delta x}&= 0,\quad for\quad a > 0, \end{aligned}\end{aligned}$$

21 $$\begin{aligned} &  \begin{aligned} \frac{u^{k + 1}_{i} - u^{k}_{i}}{\Delta t} + a\frac{u^{k}_{i - 1} - u^{k}_{i}}{\Delta x}&= 0,\quad for\quad a < 0. \end{aligned} \end{aligned}$$

The variable *a* represents the velocity at which blood propagates. A finite-difference stencil is used to discretise the system. When the blood flows from left to right, i.e., the velocity term in the advection-decay equation is positive, a backwards difference is used. For the opposing scenario, a forwards difference is computed instead (Patankar 2018). The scheme is selected because the definition of the numerical model explicitly caters for the direction in which substances propagate (Hirsch 2007). The spatial domain is discretised as a one-dimensional grid of length *L*, where *L* refers to the vessel’s length. The distance between grid points, denoted by $\Delta x$, is given by $\frac{L}{M}$, where *M* refers to the number of grid points in the domain. We can therefore define an *x*-coordinate on the domain as $i \Delta x$, where $i = 0, 1,\ldots , L$. As per the previous model, $\Delta t$ refers to the constant time step used for the scheme. Since $v > 0$, we apply equation (20) to Eq. (5) and obtain the following numerical scheme (Patankar 2018):

22 $$\begin{aligned} \frac{n^{m + 1}_{i} - n^{m}_{i}}{\Delta t} + v \frac{n^{m}_{i} -n^{m}_{i - 1}}{\Delta x} - \alpha n^{m}_{i} = 0. \end{aligned}$$

We simulate equation 3.1 using this discretization by setting $M = 10$ as it provides stability and sufficient accuracy. The numerical scheme is stable provided the Courant-Friedrichs-Lewy condition (CFL) is satisfied (Hirsch 2007). This implies the following condition is met:

23 $$\begin{aligned} c = \left| v \frac{\Delta t}{\Delta x} \right| \le 1. \end{aligned}$$

## Simulation of Metastasis

We first compare the output of the simulation against existing metastasis models. The output we receive is qualitatively similar to existing models. Cancer cells diffuse in a radially symmetric manner. We also note similar behaviour to the metastasis models established by Franssen (2019). We initiate cancer cell growth in the gut.

**Fig. 6:**
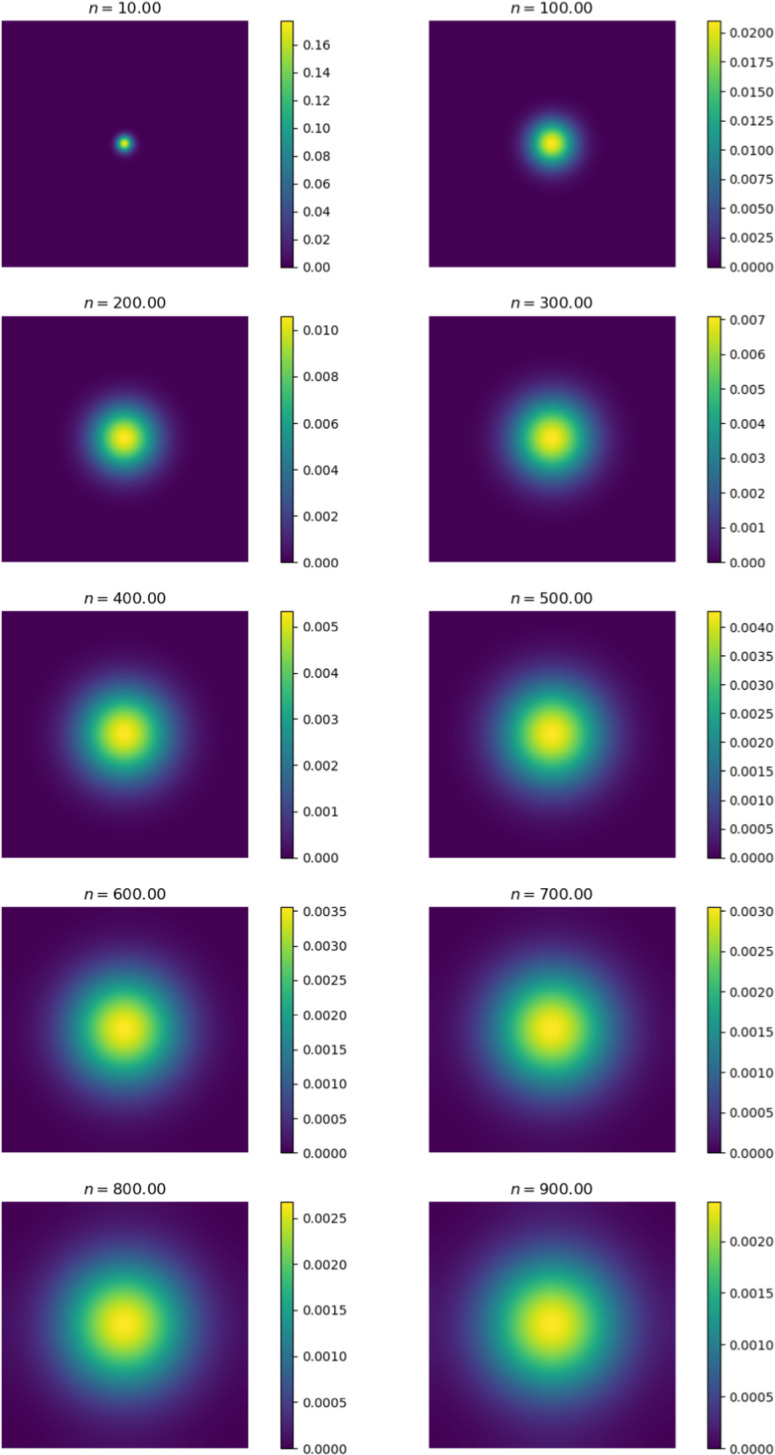
Change in cancer cell distribution in primary site (gut) (colour figure online)

**Fig. 7:**
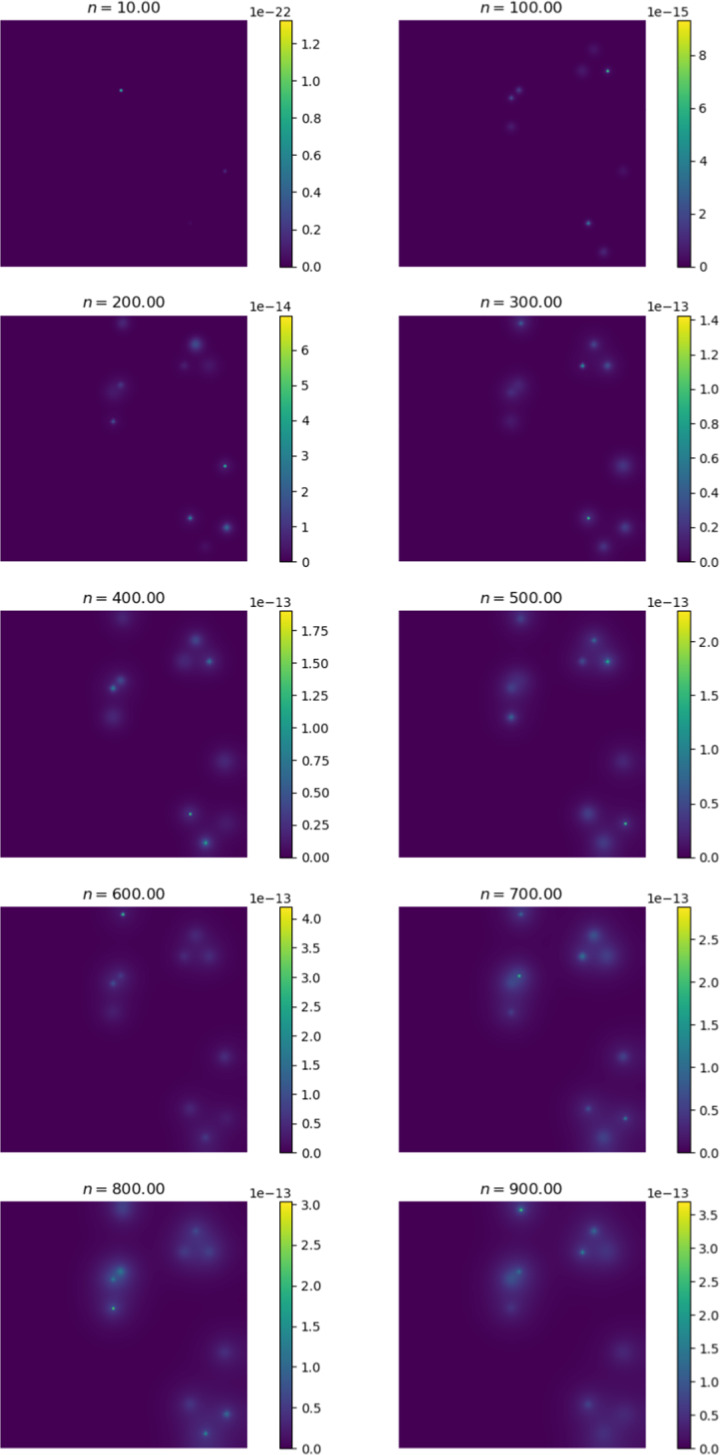
Change in cancer cell distribution in metastatic site (liver) (colour figure online)

Figure 6 shows the change in the distribution of cancer cells between various iterations of the simulation. At $n = 0$, we observe the initial configuration of the simulation. We note that the cells have diffused in a radially symmetrical manner at higher concentrations. The corresponding Fig. 7 shows the distribution of cancer cells in the liver. This site is selected as it contains the highest proportion of cancer cells outside of the primary site. Here we note the formation of micro-tumours as they proliferate in their new environment. We observe that the tumours increase in concentration as the simulation progresses. The high concentration of cancer cells in the liver is in line with clinical observations (Valderrama-Treviño 2017). The finding is expected because blood from the gut must pass through the hepato-portal vein and into the liver. Since this structure is captured in the simulation, we experience similar results.

**Fig. 8:**
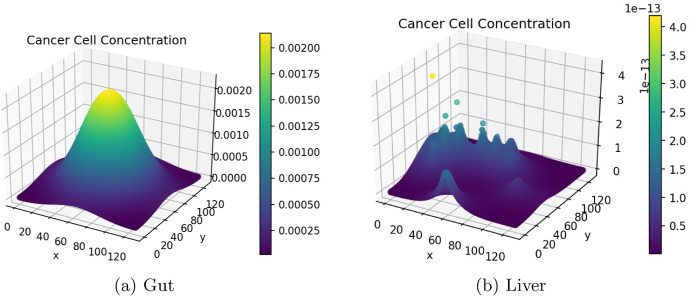
Cancer cell concentration in the gut and liver (colour figure online)

The cancer cell concentration in the gut and liver is visualised in 3-dimensions in Fig. 8. This visualisation effectively displays the growth and diffusion of the primary tumour in the primary site and the formation of successful micro-tumours in the secondary site.

**Fig. 9:**
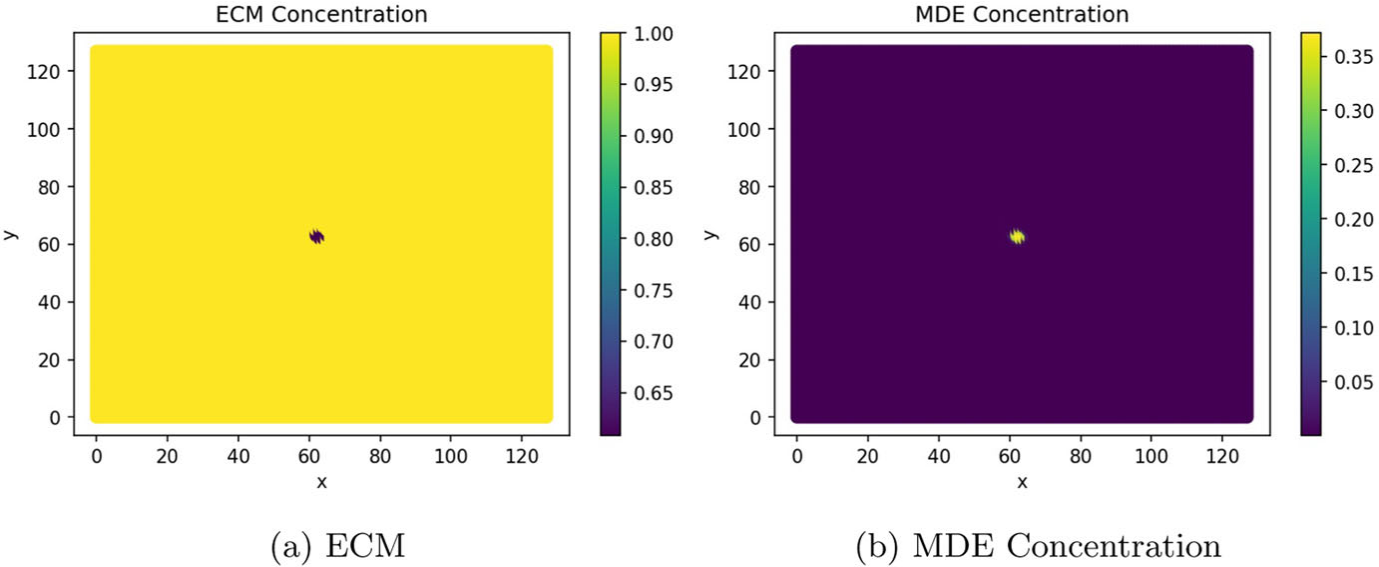
ECM and MDE concentration in the gut after 1000 iterations (colour figure online)

**Fig. 10:**
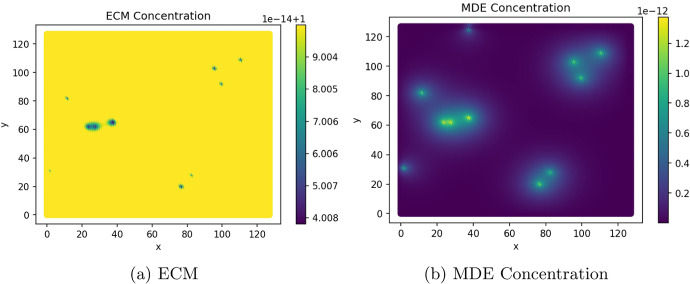
ECM and MDE concentration in the liver after 1000 iterations (colour figure online)

Figures 9 and 10 show the ECM and MDE concentration in the gut and liver respectively. An important observation from Fig. 8 is the small scale of the cancer cell concentration. To account for this, we first note that the growth we achieve on primary sites is qualitatively similar to the growth observed in other works, such as the work conducted by Pera et al. (2019). The results have a smaller scale because of the metastasis dynamics we introduced, as primary sites lose some cells to extravasation. The proliferative behaviour on secondary sites is aligned to what we anticipate based on the behaviour of the primary site—particularly, conical peaks that begin to form micro-tumours as the simulation progresses. This growth is also qualitatively similar to the views from Franssen (2019). It differs on a quantitative level because much of the dynamics in Franssen (2019) was modelled using discrete techniques. For these reasons—we infer that the simulation is appropriate to explain qualitative behaviour of metastasis despite the small scale. It is, therefore, not used to predict exact values (like the exact concentrations of cells after a certain amount of time)—but rather to elucidate the more mechanistic aspects of metastasis; hence we express quantitative differences as proportions.

The behaviour displayed in Fig. 9 shows that the degradation of the ECM is aligned to the production of MDEs. We experience the most degradation in the region’s centre as the cancer cells are primarily concentrated there. In Fig. 10, the ECM and MDE degradation align with the sites of micro tumours.

**Fig. 11:**
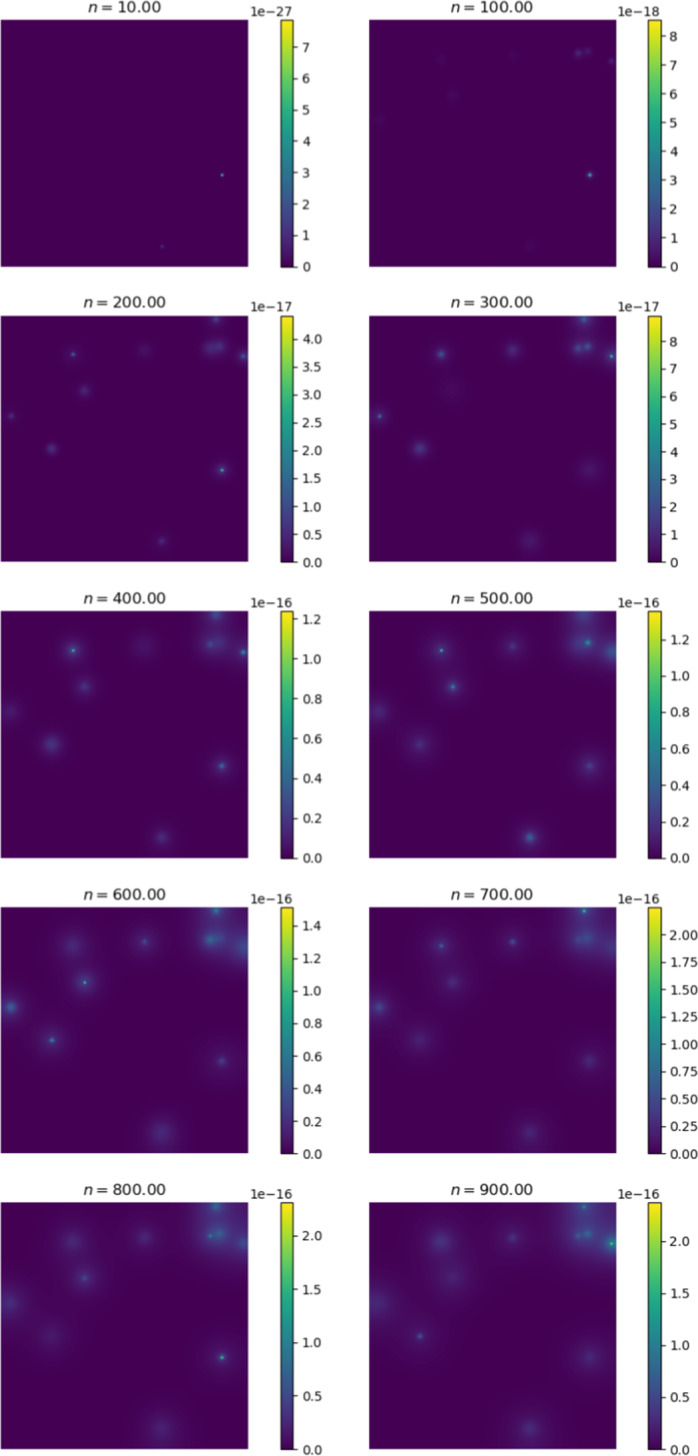
Change in cancer cell distribution in metastatic site (lung) (colour figure online)

**Fig. 12:**
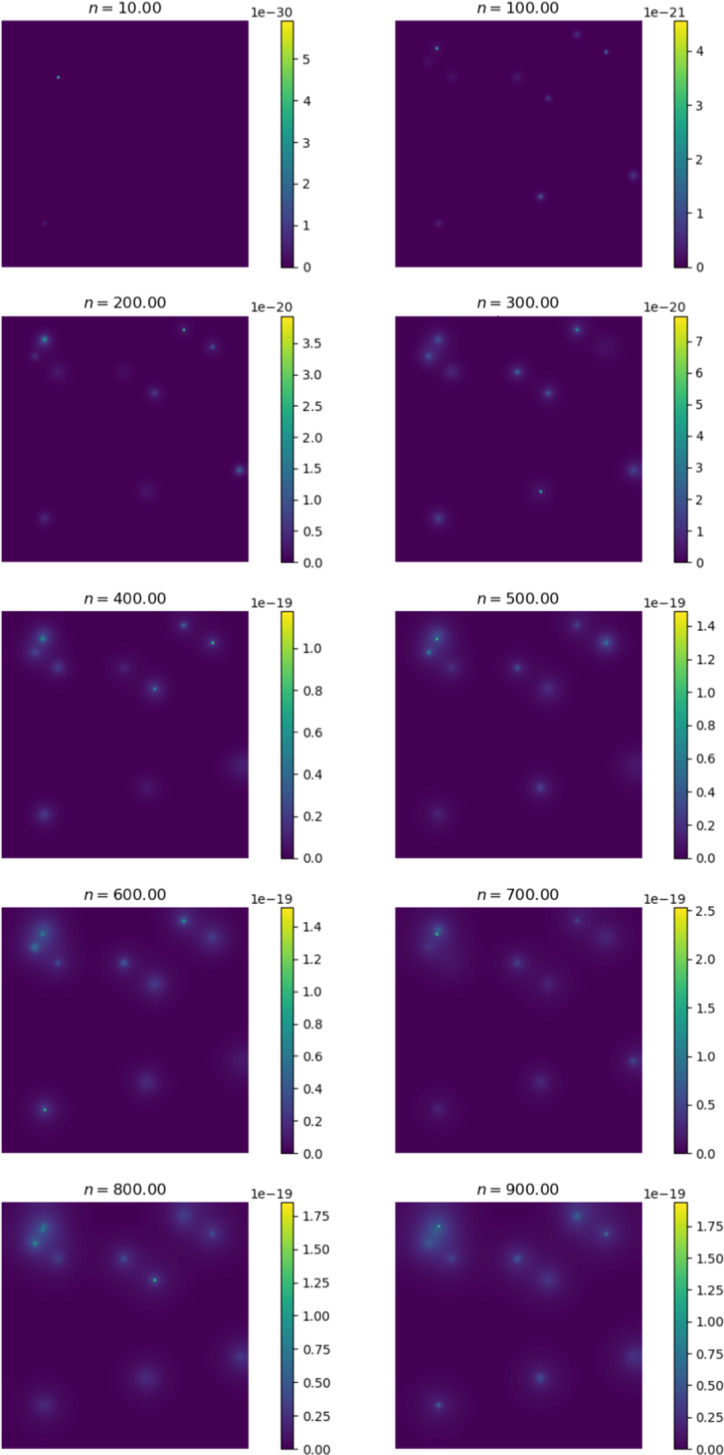
Change in cancer cell distribution in metastatic site (brain) (colour figure online)

**Fig. 13:**
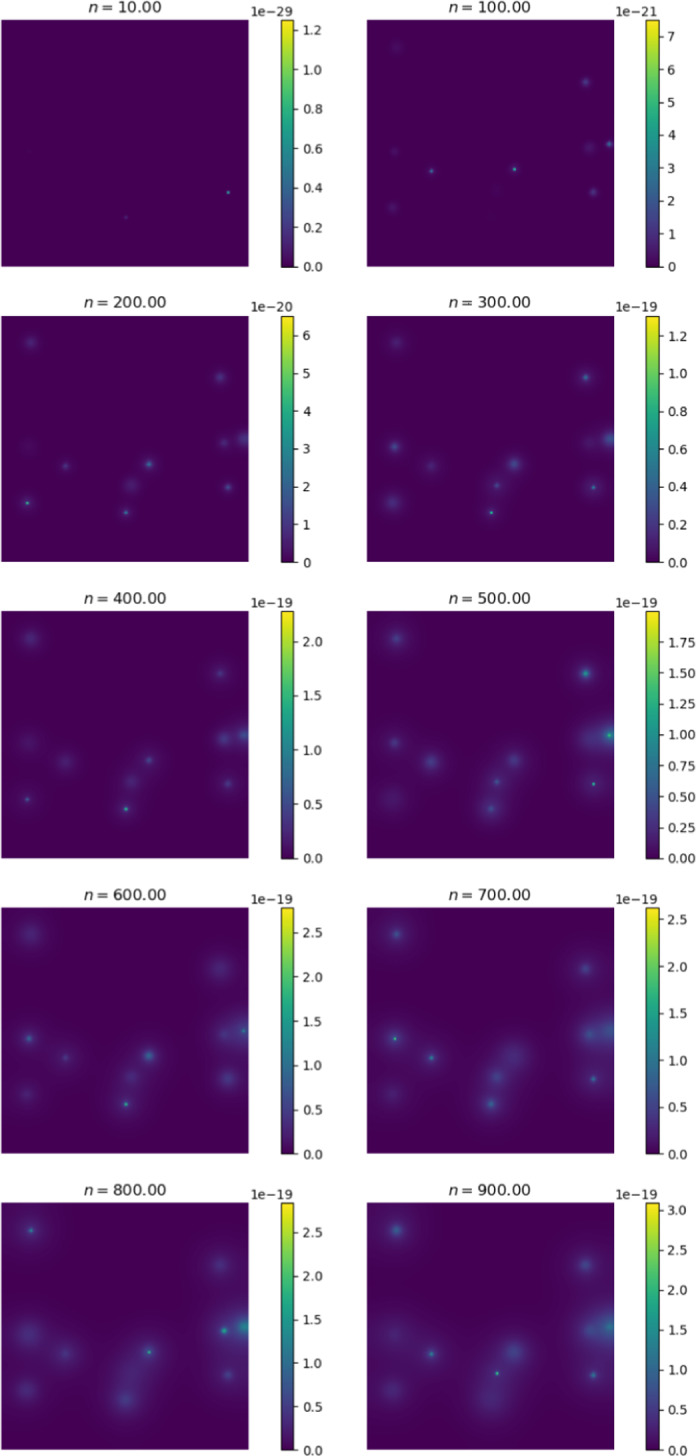
Change in cancer cell distribution in metastatic site (kidney) (colour figure online)

The following series of figures shows the concentration of cancer to other organs in the system, namely, the lungs, brain and kidney. Notably, each organ contains some micro-tumour development mainly because of how the model is constructed. Cells are not prevented from travelling to other locations; a small proportion migrates to other sites based on the quantity of blood arriving at the organ. However, in some organs, this value is minimal; this can be interpreted as a lower probability of metastasis (Figs. 11, 12, 13).

Note that while each figure shows the presence of some micro-tumours, the scale is not the same for each organ. Therefore, despite having qualitatively similar behaviour, the relative concentration of cells differ.

While the model has clinically relevant results for the gut, the potential spread from cancers originating in other organs must be considered to determine the model’s overall performance. We, therefore, analyse data sourced from the Human Cancer Metastasis Database (HCMDB) (Zheng 2018). Our focus is solely on cancers arising in and metastasising to the organs under study. We use the data to determine the common metastasis sites for each organ and determine whether the model’s predictions align with these findings.

When analysing the HCMDB data, we first filter the primary and secondary sites for only the organs under study. After that, for each primary site, we determine the percentage of secondary metastases formed at each secondary site. Regions such as the small intestine, stomach and colorectum are all classified as cancer of the gut to align with the cardiac anatomy model in Iaizzo (2010). The results are summarised in the proceeding table (Table 3).

**Table 3:** Distribution of metastatic sites

Primary site	Secondary site				
	Brain (%)	Gut (%)	Kidney (%)	Liver (%)	Lung (%)
Brain	100	0	0	0	0
Gut	0.16	0.25	0	90.76	8.83
Kidney	0	0	0.99	8.42	90.59
Liver	0	0	0	8.89	91.11
Lung	100	0	0	0	0

**Fig. 14:**
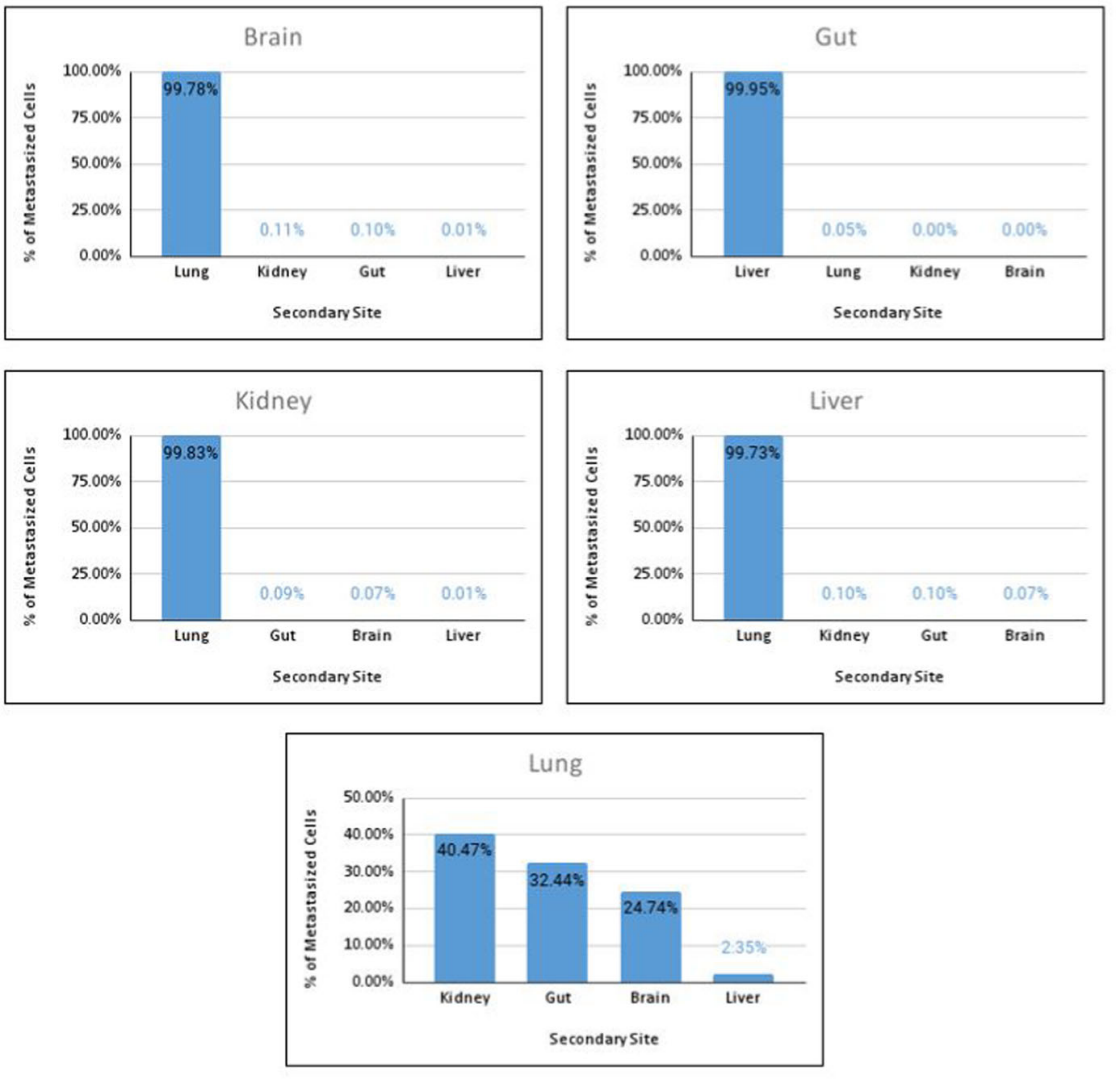
Percentage of cancer cells found in secondary sites for each of the organs under study

We now contrast the information from the preceding table with the outputs generated by the model. The results are expressed by counting the number of cells in each secondary location and expressing this as a percentage of the total number of cells. This information is summarised in Fig. 14. For each primary site, we compare the findings from the simulation against the HCMDB data. Next, we contextualise these results with the relevant tumour pathology; this allows us to draw inferences on the model’s performance and, where possible, make conclusions on the role of hematogenous metastasis for cancers originating in the selected primary sites.

The HCMDB analysis notes that cancers from the gut primarily metastasise to the liver. Tumours originating in the kidney and the liver tend to metastasise to the lung. In all records of interest, lung cancer spreads to the brain. Of particular interest is the brain, which displayed local metastasis for all of the observations in the data set. The simulation displays similar results for the gut, kidney and liver and conflicting views for the lung and brain. We discuss both the contradictory and complementary results.

When selecting the brain as the primary site, the simulation shows that most metastatic cells colonise the lung. This finding can be ascribed to the large volume of blood the lungs receive from venous circulation. However, the HCMDB data shows that the overwhelming majority of brain cancers produce only local metastases. A review of the literature confirms that cancers of the brain, or central nervous system (CNS), rarely produce secondary metastases. This phenomenon is attributed to a host of reasons. Firstly, the brain lacks specific ECM components which facilitate the migration of cancer cells into the bloodstream; thus, while cancer can proliferate, it cannot easily invade (Pansera and Pansera 1992). Another explanation for the observed rarity of brain metastases is the low survival rate of brain cancer. Patients typically lead much shorter lifespans; thus, cancer does not have sufficient time to metastasise (Mentrikoski 2008). Both these factors are not considered in the model, as, aside from varying the type of diffusive behaviour at each organ, we do not consider organs for which diffusion is complex. The lifespan of patients is also excluded from our model parameters. We can thus conclude that the brain favours neither hematogenous nor lymphatic metastases as the primary method of spread.

As discussed earlier in the section, our model predicted the liver as the site that receives the largest concentration of metastatic cells from the gut. After the liver, the lung receives the most significant proportion of tumour cells. These findings align with the HCMDB data, as the liver is the most common metastatic site for colorectal cancers, while the lung is the second most prevalent site. While our model does imply the possibility of metastatic lung cancer, it does not occur independently of liver metastases. In reality, isolated lung metastases are relatively rare (Tan et al. 2009). In our analysis, they represent $8.83\%$ of all metastatic colon cancers. One possibility for this discrepancy lies in our grouping of cancers originating in the gut. Cancers of the rectum, as opposed to the colon, are more likely to result in lung metastasis (Tan et al. 2009). This observation can be attributed to the ‘seed and soil’ hypothesis. From which We can infer that the lung is likely a favourable soil for the cancer cells originating in the rectum. The colonisation of the lungs may also arise from lymphatic metastasis. Cancer cells from the lymph nodes enter venous circulation via the subclavian vein; consequently, the first capillaries the cells come into contact with is in the lung Naxerova (2017). From our analysis, we may conclude that hematogenous metastasis is likely the cause of secondary metastasis to the liver. It is unlikely that hematogenous metastasis is solely responsible for isolated metastases to the lungs, as other metastatic mechanisms are probably at play.

Primary lung cancers generally metastasise to the brain, bone, and adrenal glands (Popper 2016). Since the bone and adrenal glands are beyond the scope of this study, we removed them from the data set and the simulation. Our simulation identified many cancer cells in all organs except the liver; this is in line with the estimated cardiac output to these organs displayed in Fig. 1. The Kidneys received the most blood, at $22\%$, with the gut following closely behind at $21\%$. The brain receives $14\%$ and the liver $6\%$. Lung cancer-specific data, however, shows that the most common metastatic sites for the lung are the CNS and the liver, with $47\%$ and $35\%$ prevalence in the data (Riihimäki 2014). Although this has been shown to differ based on the histology of the cancer (Milovanovic et al. 2017). The liver micro-environment may be hospitable to lung cancer. Alternatively, the spread may be driven by the lymph nodes. Of particular importance, however, is metastasis to the brain. The brain is the most common secondary site for lung cancer. The mechanisms which drive this particular spread are not fully understood. One proposition for the high incidence of secondary brain tumours is the seed and soil hypothesis. The brain is likely a favourable soil for lung cancer (Ebben Johnathan and Ming 2016). Unfortunately, the model does not capture the complexities of the seed and soil hypothesis and the nuances of differing micro environments; this shows that these factors play a crucial role in the metastatic cascade, as it supersedes the results we anticipate from a purely hematogenous spread.

Cancers of the liver metastasise mainly to the lung. With the brain being the least likely secondary location (Wenrui 2017). These findings show some alignment to the model, as the lung receives the highest proportion of cells while the brain receives the lowest. This finding shows good alignment with clinical data and implies that hematogenous spread is a strong driver for liver metastases. Based on our model, The kidney and gut receive the second and third highest proportion; this, however, is not aligned with the HCMDB, as no metastases to these sites were identified. It is worth noting that the model predicted very small proportions in these organs.

For the kidney, our model produced the most number of metastases in the lung, followed by the gut, brain and liver. In the HCMDB data, the kidney metastasizes most frequently to the lung and liver. The brain is also a common secondary site for cancer of the kidney (Sivaramakrishna 2005), despite being absent from the HCMDB data we analysed. Venous blood from the colic vein drains directly into the hepato-portal vein; this potentially explains why isolated liver metastases are common for kidney cancer patients. These veins were excluded from the simulation, and hence this behaviour is not reflected. Since this is still a vascular pathway, we can conclude that hematogenous spread predicted by the simulation could play a vital role in both liver and kidney metastasis. It is important to note that lymphatic spread may also be a driving factor for lung metastases.

## Varying Blood Velocity

In this section, we review the effects of blood velocity on the process of metastasis. The velocity of blood in each vessel is varied across biologically realistic ranges. We run the simulation using the lower and upper bounds for velocity. For consistency, we initialise cancer in the gut. These values are compared against the cancer cell concentration when using the average blood velocity, allowing us to determine the net effect of varying the velocity. The proceeding table contains the lower, average, and upper values of blood velocity in the relevant veins and arteries. Where values could not be sourced for velocity, we selected values within a reasonable range for blood velocity, as per some of the velocities identified in Gabe (1969), while adhering to the CFL condition. The qualitative dynamics of the system were not appreciably affected by the choice of these values (Table 4).

**Table 4:** Blood velocity ranges per blood vessel

Vessel name	Lower bound (cm/s)	Average (cm/s)	Upper bound (cm/s)	Reference
Pulmonary Artery	10	33.5	57	Gabe (1969)
Pulmonary Vein	10	15	20	N/A
Renal Artery	40	50	60	Al-Katib (2017)
Renal Vein	10	15	20	N/A
Carotid Artery	80	100	120	Brant (2001)
Jugular	10	15	20	N/A
Abdominal Aorta	24	28	32	Stein (1979)
Hepato-portal Vein	20	30	40	Iranpour (2016)
Renal Vein	10	15	20	N/A

**Fig. 15:**
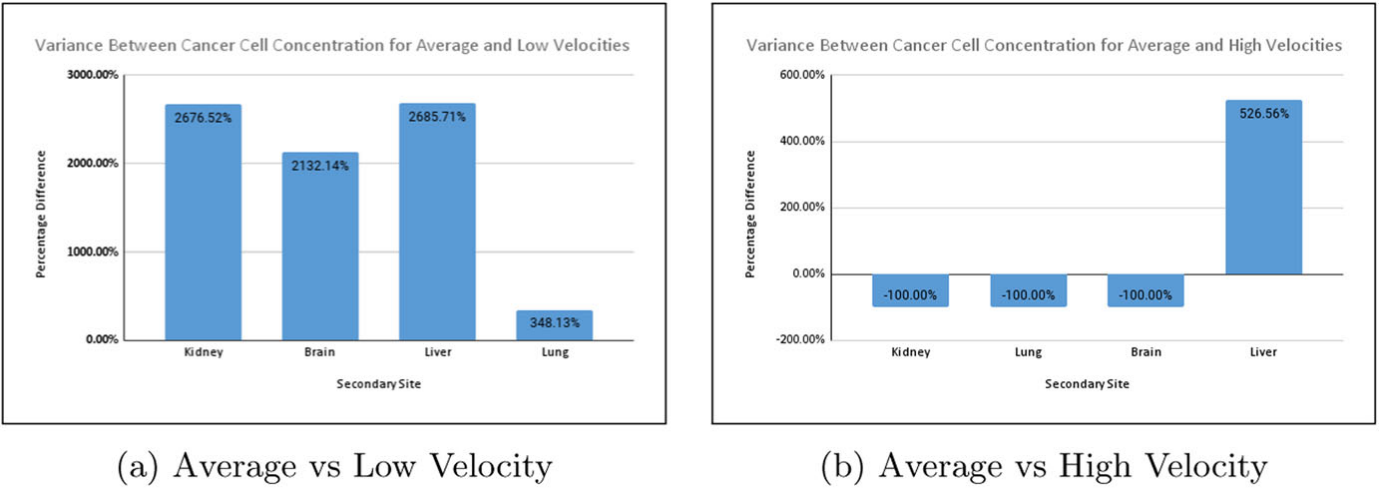
Difference in cell concentration for lower and higher velocities

Running the simulation using the lower, average and upper velocities produced the results in Fig. 15. We express these values as the percentage difference between low and high-velocity outputs and the average velocity output. This comparison gives us a clear indication of the impact of velocity in the simulation.

From these observations, we note that cancer did not metastasise to the lungs, kidney, and brain for higher velocity ranges, as there was a $100\%$ decrease in cell concentration on those organs. There was, however, a very significant increase in cell concentration in the liver. At lower velocities, there was a substantial increase in the cell concentration at all locations. The cell concentration on the gut remained relatively consistent across each scenario.

In most cases, the model predicted an inverse relationship between blood velocity and cancer cell concentration, except for the liver at a high blood velocity. This discrepancy is possibly due to the proximity of the liver to the gut. The relationship between blood velocity and cancer spread is not well defined, and thus corroborative evidence is challenging to procure. These findings indicate that sustained variations in blood velocity can impact the spread of cancer. Since the simulation predicts that using higher velocities significantly lowers the metastatic potential of cancer, we can assume that this may have some bearing on metastatic inefficiency.

**Fig. 16:**
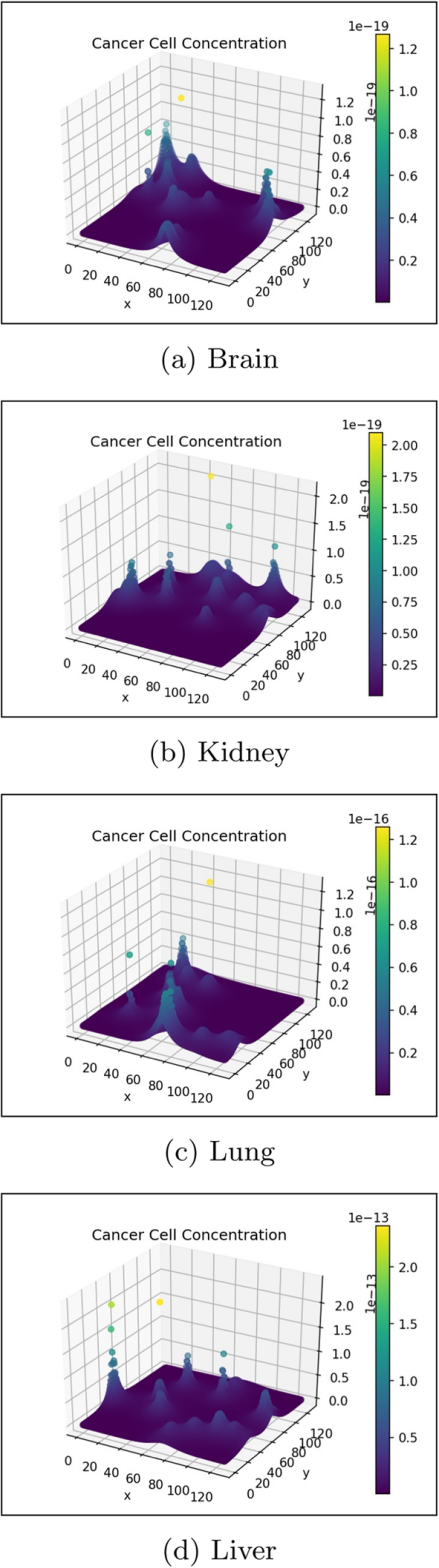
Cell concentration for normal velocities (colour figure online)

**Fig. 17:**
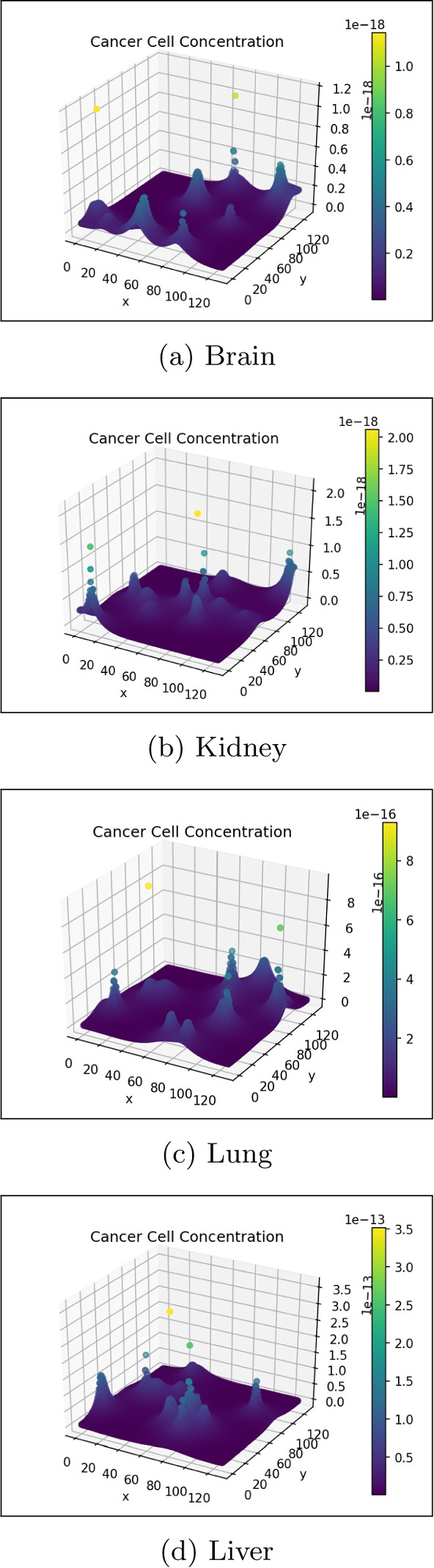
Cell concentration for lower velocities (colour figure online)

**Fig. 18:**
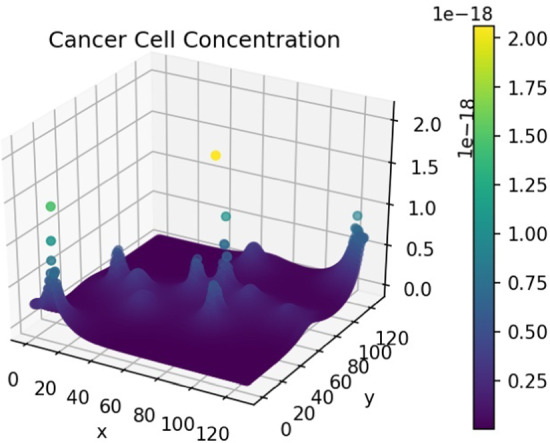
Cell concentration in liver at higher velocities (colour figure online)

Figures 16, 17 and 18 provide a 3-dimensional view of the micro-tumours that formed at each organ for the various blood velocities. This shows, qualitatively, the impact velocity can have.

## Varying Diffusive Conditions

Thus far, we assumed that cancers exhibit isotropic diffusion. This assumption is not always valid, as some cancers diffuse anisotropically. For example, certain brain cancers—known as gliomas (Roniotis 2011). To study this effect on metastasis, we initialise cancer in a primary site and change the diffusion tensor to allow for anisotropic diffusion. The cancer cell concentration is aggregated and contrasted against the isotropic case. Similar to the previous sections, we initialise cancer in the gut. To achieve anisotropic diffusion, we set the value of *a* in the diffusion tensor to 0.1. This increases diffusion 10-fold in the *y*-direction (Pera et al. 2019).

**Fig. 19:**
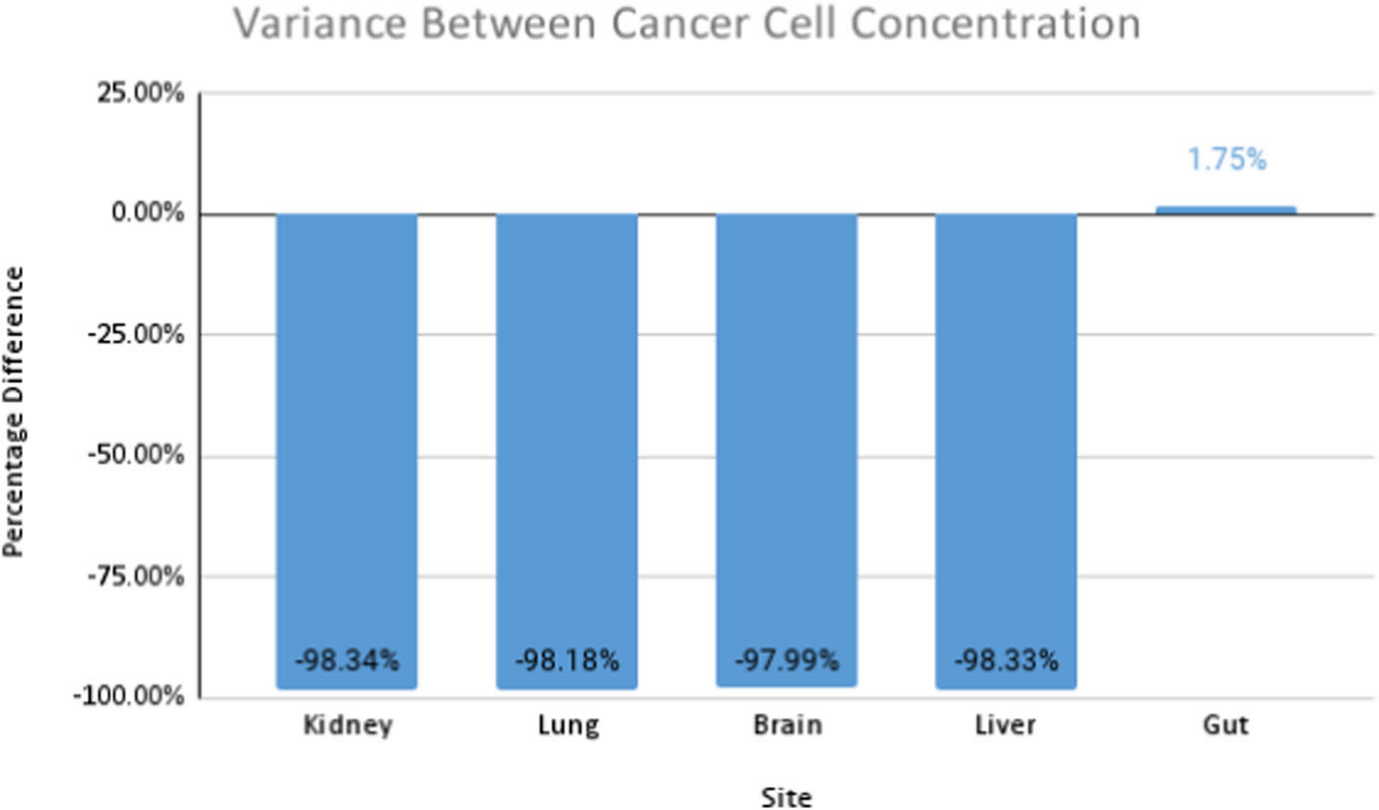
Percentage difference between cancer cell concentration in all locations for anisotropic and isotropic diffusion

**Fig. 20:**
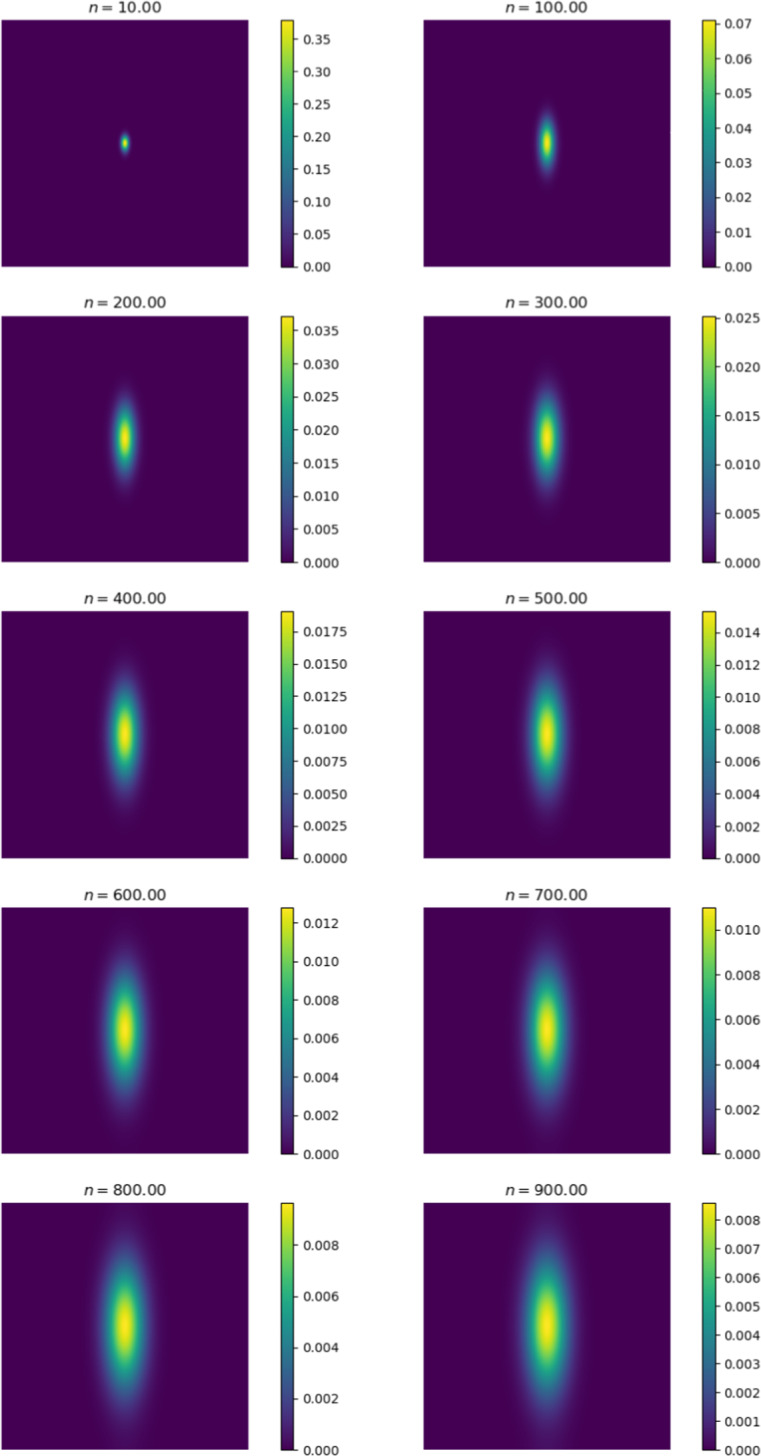
Change in cancer cell distribution in primary site (gut) (colour figure online)

**Fig. 21:**
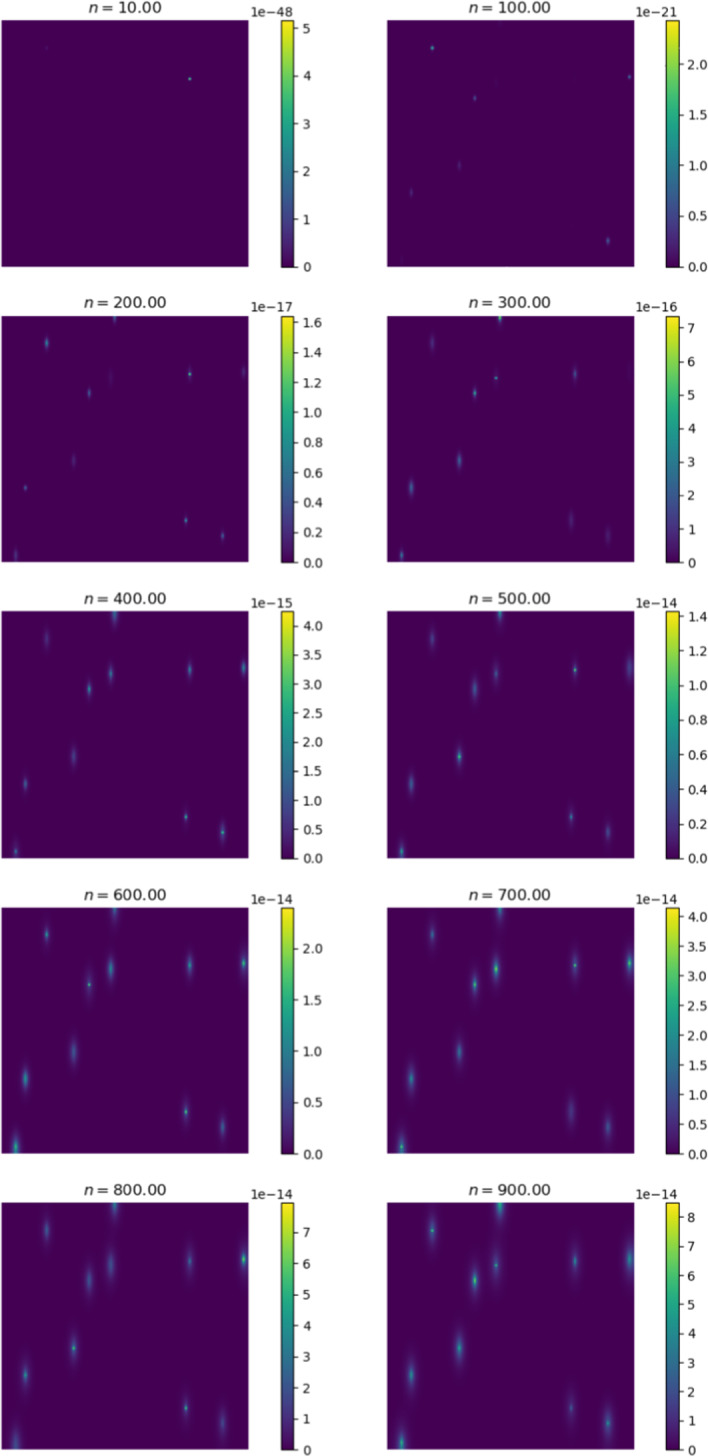
Change in cancer cell distribution in metastatic site (liver) (colour figure online)

Figure 19 displays the difference in cell concentration for both types of diffusion. There is a consistent decrease of around $98\%$ of cancer cells in secondary locations for anisotropic diffusion. The primary site displays a slight increase in cell concentration. Figures 20 and 21 provide a general diffusion pattern for anisotropic diffusion on a primary and secondary site. We focus on the gut and liver, as the liver’s behaviour indicates the general diffusive behaviour we observed in all the secondary sites.

**Fig. 22:**
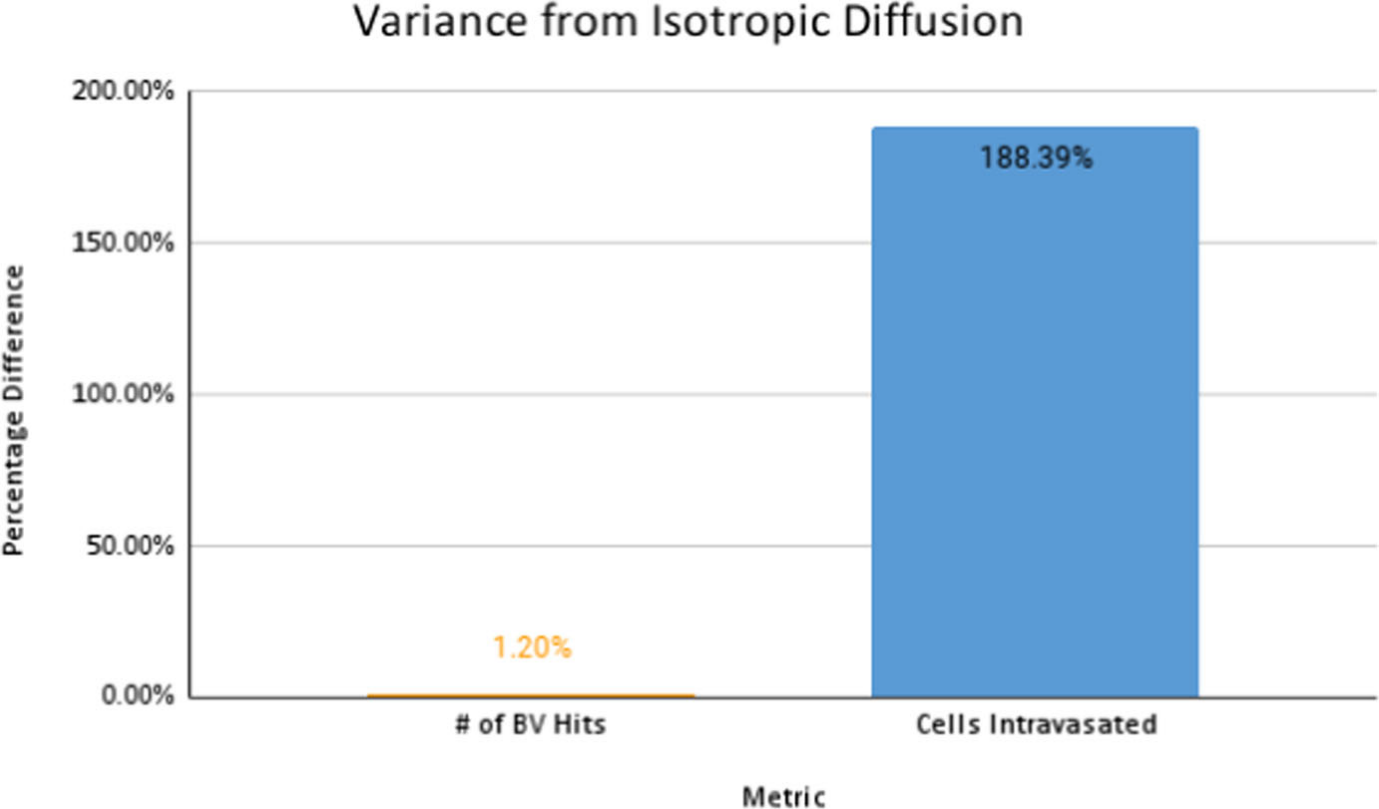
Percentage difference between the number of blood vessel hits and cells intravasated for anisotropic versus isotropic diffusion

In Fig. 20 the reinforced diffusion in the y-direction is clear. To understand why there is a decrease in cell concentration, we determine the number of intravasated cells and the number of blood vessels that have been reached for both cases. We focus on these as the diffusive behaviour directly impacts the number of cancer cells that arrive at the vessels and the number of times the vessels are reached. The general behaviour is shown in Fig. 22.

We note there is a slight increase in the number of times a blood vessel is reached and a significant increase in the number of intravasated cells. This behaviour is likely due to the increased diffusion in the *y*-direction. The cancer cells can reach blood vessels located at the upper and lower quadrants of the region. Since cancer still diffuses in the *x*-direction, some intravasation occurs at blood vessels in these regions. The slight increase in blood vessel hits and the large concentration of cells that reach the blood vessels in the upper and lower quadrants are potentially responsible for the considerable increase in extravasated cells. Since we note an increase in both these metrics, we can conclude that these factors do not decrease cell concentration in the secondary sites.

These findings deduce that anisotropic diffusion may result in a slightly higher concentration of cancer cells in the primary site but a lower concentration of cells in the secondary site. This type of diffusion also causes a larger proportion of cells to undergo intravasation. Anisotropic tumours can thus be considered less efficient for metastasis. Secondary sites appear to be a hostile environment for tumours that diffuse anisotropically; this is consistent with the behaviour of gliomas, as they do not generally produce secondary metastases. Diffusive behaviour can be responsible for metastatic inefficiency, a factor that is not generally considered.

## Conclusion and Discussion

This research aimed to develop a holistic model of metastasis. The impetus for this model is to encapsulate the process of metastasis and determine the how different patient and cancer specific characteristics impact metastasis. In particular, the velocity of blood and diffusive behaviour of cancer.

We find that the simulation aligns with previous models for cancer growth on the primary site. We also observed a substantial correlation with the secondary sites for cancer of the gut and liver against clinical data. There were some deviations for the brain, lung and kidney. These deviations are due to the unique environment of the brain, potentially the ‘seed and soil’ hypothesis for the lung and some vasculature we excluded for the kidney. Despite the excluded vasculature, we note that the model does show some good alignment with the clinical data. The model allows us to understand the impact of hematogenous metastasis for the various organs under study. From these results, we can infer that hematogenous metastasis is a primary driver for metastasis of the gut, liver and potentially the kidney. Metastasis is very unlikely for the brain, while other factors are more critical for cancer spread from the lung. These factors include the above-mentioned ‘seed and soil’ hypothesis and potentially lymphatic spread.

We found an inverse relationship between blood velocity and cancer cell concentration concerning the second of our research questions. This case holds except for when cancer originates in the gut and spreads to the liver at high blood velocities. This behaviour is possibly due to the proximity of the gut and liver. Since there is a lack of clinical data, we cannot contrast the model against practical observations. We can conclude that it is likely that blood flow velocity does affect cancer spread. The blood velocity use case also demonstrates how this framework can simulate patient-specific information.

The diffusive behaviour of cancer clinical significance because some cancers display anisotropic diffusion. Here the model predicted an overall decrease in the cancer cell concentration at the secondary sites. This behaviour holds for some cancers that diffuse anisotropically, particularly gliomas of the brain. We have noticed a slight increase in cancer cells in the primary site and a sizable increase in intravasation from the primary site. This behaviour shows us the effect of varying diffusion on the overall spread. To our knowledge, the existing body of research on mathematical oncology has not explored the impact of anisotropic diffusion on metastasis.

The model does provide a good framework for exploring how some of these factors, which may be patient or case-specific (such as the diffusive nature of the tumour), can be simulated for mechanistic purposes. Overall, this research allows practitioners to study the global effects of cancer in a manner that has hitherto been difficult to simulate. However, our focus on the global impact does restrict the histological factors we can consider at a smaller scale. These exclusions can harm accuracy, as we may inadvertently omit potentially important information. For example, a more appropriate treatment of blood vessel concentration at each node would consider the organ system that the node represents. This is left as a possible extension.

We have introduced a framework that encapsulates the global behaviour of cancer. This methodology can be expanded on to include other behaviour at the cellular level; for example, cancer cells often intravasate as clusters. These clusters can impact the process of intravasation and the survival probability of cells in the vasculature (Franssen 2019).

Furthermore, other means of transportation, particularly transport through the lymph, can significantly advance the model’s scope. We use a simple abstraction of blood dynamics in this model—further research can expand this to include more appropriate fluid dynamics. In addition to fluid dynamics, there are other biological factors cancer experiences in the bloodstream which we have ignored—for example, the immune response that the patient expresses. Another interesting extension would be the study of more complex diffusive phenomena. This study considered anisotropic diffusion along a single axis, this can be extended to more unorthodox tumour geometries. Perhaps most interesting is the opportunity to include more patient-specific information. For example, a patient’s unique vessel permissivity can determine the likelihood of the cell to intravasate.

## Data Availability

The Human Cancer Metastasis data used to support the findings of this research are available as open data via the Human Cancer Metastasis Database (HCMDB) repository: http://hcmdb.i-sanger.com/index, http://hcmdb.i-sanger.com/index.
